# Designing a multi-epitope vaccine against yellow fever virus using immunoinformatics approaches

**DOI:** 10.1515/med-2026-1453

**Published:** 2026-06-18

**Authors:** Muhammad Naveed, Muhammad Asim, Tariq Aziz, Muhammad Toheed, Aroosa Athar, Muhammad Nouman Majeed, Ayaz Ali Khan, Maha Alharbi, Ashwag Shami, Maher S. Alwethaynani, Mai M Almsaud, Fakhria A. Al-Joufi

**Affiliations:** Department of Biotechnology, Faculty of Science and Technology, University of Central Punjab, Lahore, Pakistan; Laboratory of Animal Health, Hygiene and Food Quality, University of Ioannina, Arta, Greece; Department of Biotechnology, University of Malakand Chakdara, Dir Lower, KPK, Pakistan; Department of Biology, College of Science, Princess Nourah bint Abdulrahman University, P.O. Box 84428, Riyadh 11671, Saudi Arabia; Department of Clinical Laboratory Sciences, College of Applied Medical Sciences, Shaqra University, Alquwayiyah, Riyadh, Saudi Arabia; Department of Biology, College of Science, Imam Mohammad Ibn Saud Islamic University (IMSIU), Riyadh, Saudi Arabia; Department of Pharmacology, College of Pharmacy, Jouf University, Aljouf, Saudi Arabia

**Keywords:** yellow fever virus, multi-epitope vaccine, immunoinformatics, toll-like receptors, molecular docking, molecular dynamics simulation

## Abstract

**Objectives:**

Yellow fever virus (YFV) is a mosquito-borne pathogen causing severe hemorrhagic fever in tropical and subtropical regions. This study aimed to design and evaluated a multi-epitope subunit vaccine against yellow fever virus using immunoinformatics and computational approaches.

**Methods:**

The yellow fever virus envelope glycoprotein was selected as the target antigen. Antigenic, non-allergenic, and non-toxic B-cell, MHC-I, and MHC-II epitopes were predicted and assembled into a vaccine construct using linkers and β-defensin-3 as an adjuvant. Structural modeling and validation were performed using AlphaFold3 and quality assessment tools. Molecular docking with TLR2 and TLR8 was conducted using ClusPro, followed by molecular dynamics simulations to assess structural stability. Disulfide engineering was applied to enhance rigidity, immune simulation was performed to predict host immune responses, and *in silico* cloning was carried out using the pBR322 vector.

**Results:**

Six conserved epitopes with strong antigenic potential were identified. The vaccine construct showed favorable docking interactions with TLR2 and TLR8, yielding ClusPro weighted interaction scores of −1105.52 and −1152.9, respectively. Molecular dynamics simulations revealed structural stability, supported by stable RMSD and compact radius of gyration profiles. Immune simulation indicated robust humoral and cellular immune responses.

**Conclusions:**

The designed multi-epitope vaccine showed promising immunogenic and structural properties, supporting its potential as a potential vaccine candidate against yellow fever virus. However, experimental validation through *in vitro* and *in vivo* studies is required.

## Introduction

Yellow fever virus (YFV) is a mosquito-borne flavivirus and belongs to the family *Flaviviridae*. It causes yellow fever, a serious hemorrhagic disease endemic to tropical and subtropical regions [1]. Aedes and Haemagogus species of mosquitoes mainly transmit this disease, whereas non-human primates play an important role in maintaining the virus in sylvatic transmission cycles [2]. YFV possesses a single-stranded positive-sense RNA genome [3]. It encodes structural and non-structural proteins required for viral replication and pathogenesis. Among these, the envelope glycoprotein (E protein) is essential, as it plays a crucial role in viral attachment to host cells and fusion of the viral envelope with the host cell membrane during entry [4]. Immunoinformatics-based approaches have been widely applied for the design and evaluation of multi-epitope vaccine candidates, enabling the identification of immunogenic epitopes and the assessment of vaccine stability, antigenicity, and structural properties [5]. During the 17th–19th centuries, yellow fever spread throughout the Caribbean and the Americas, causing recurrent outbreaks and major epidemics in several regions, including North America [6].

Symptoms of yellow fever appear after an incubation period of 3–6 days [7]. The disease follows three distinct phases [8]. In the acute phase, a person experiences fever, chills, headache, muscle soreness, particularly in the back, fatigue, loss of appetite, vomiting, and nausea [9]. However, individuals may recover after this acute phase [8]. Despite this, around 15 % of patients enter the toxic phase [10]. During the toxic phase, patients experience severe symptoms such as high fever, jaundice, and abdominal pain [11]. Some may suffer from kidney failure and other severe manifestations of yellow fever [12]. This may lead to multi-organ failure, characterized by shock, internal bleeding, liver dysfunction, and failure of multiple organs [13]. Severe yellow fever can be fatal, and treatment is limited to supportive care aimed at managing symptoms and preventing complications [14]. The tropical regions of Africa and South America are the highly affected by yellow fever [15]. The World Health Organization (WHO) estimates that around 200,000 cases and 30,000 deaths occur annually due to yellow fever; however, these numbers are likely underreported due to surveillance limitations. Almost 90 % of cases and deaths occur in Africa. In South America, Bolivia, Peru, and Brazil are among the affected regions, particularly in forested areas [16], 17]. The risk of transmission is further increased by global travel [18].

The 17D live-attenuated vaccine is a widely used vaccine for yellow fever and is highly effective, providing long-term immunity in most individuals [19]. However, it has several limitations that necessitate alternative vaccine strategies. The vaccine can cause life-threatening complications, including yellow fever vaccine-associated viscerotropic disease (YEL-AVD) and yellow fever vaccine-associated neurotropic disease (YEL-AND), particularly in elderly individuals [20]. Moreover, global vaccine shortages can hinder effective outbreak control [21]. In addition, limitations associated with conventional vaccines have driven the development of alternative vaccine strategies [22]. In the absence of specific antiviral therapies, disease management relies on supportive care, highlighting the need for safer and more effective vaccine strategies.

This study aims to design an *in silico* multi-epitope vaccine against yellow fever virus using immunoinformatics approaches, targeting the envelope glycoprotein as a key antigen involved in viral entry. The rationale of this study is to develop a safer and more effective vaccine candidate alternative to existing vaccines. It is hypothesized that a computationally designed multi-epitope vaccine may exhibit favorable immunogenic and structural properties, along with potential interactions with immune receptors as evaluated through molecular docking and molecular dynamics simulations. Therefore, the research question addressed is whether the designed vaccine construct can demonstrate predicted immunogenicity, structural stability, and receptor-binding potential through *in silico* analyses.

## Materials and methods

**Figure 1:**
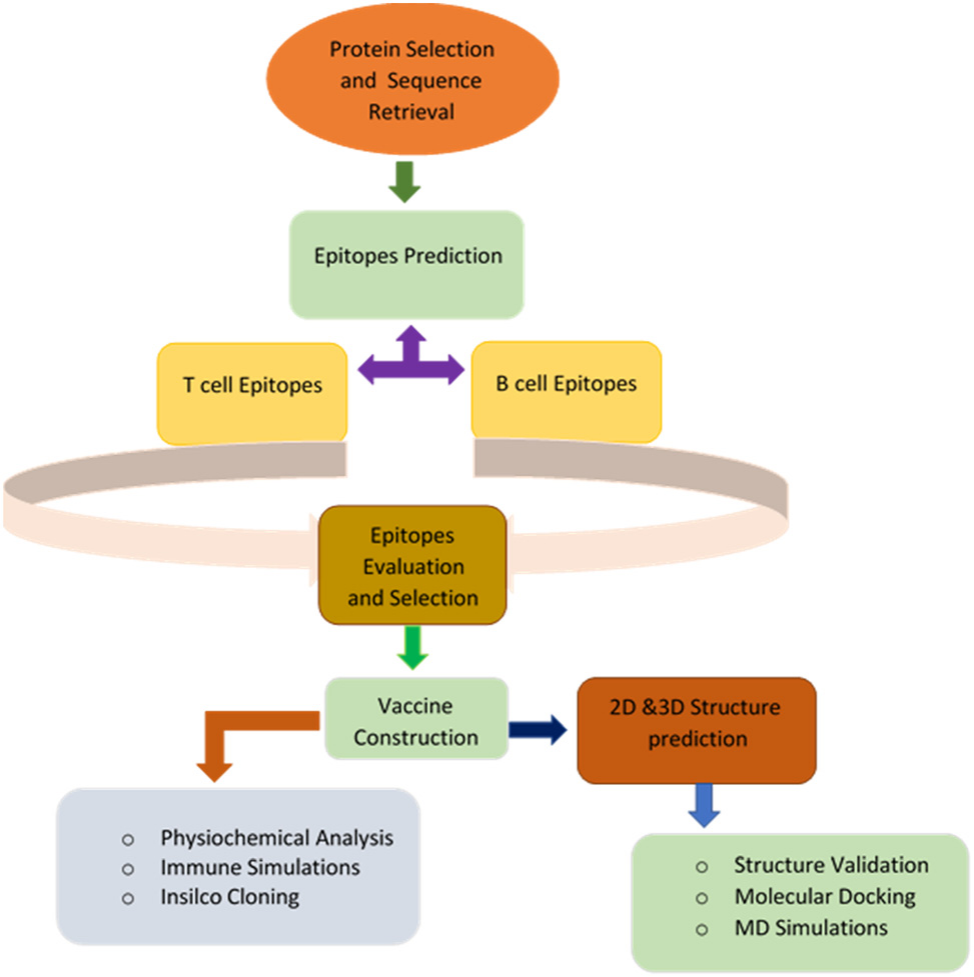
Schematic representation of the methodology for multi-epitope vaccine construction against yellow fever virus.

The overall workflow of the multi-epitope vaccine design is illustrated in Figure 1. The process begins with protein selection and epitope prediction, followed by screening and selection of B-cell and T-cell epitopes based on immunological properties. The selected epitopes were then assembled into a vaccine construct and further evaluated through structural modeling, validation, molecular docking, and molecular dynamics simulations, along with physicochemical and immune response analyses.

### Protein selection and sequence retrieval

The initial step in the design of a multi-epitope vaccine is the selection and retrieval of the target protein of a specific organism. The amino acid sequence of the selected protein of yellow fever virus was retrieved from UniProt (https://www.uniprot.org/), a well-known resource providing high-quality protein sequences and their functional descriptions [23]. The antigenic potential of the selected envelope glycoprotein was assessed using the VaxiJen v2.0 server (https://www.ddg-pharmfac.net/vaxijen/VaxiJen/VaxiJen.html), which predicts antigenicity based on an alignment-independent approach [24]. In addition, the allergenic potential of the protein was evaluated using the AllerTOP v2.1 server (https://ddg-pharmfac.net/allertop_test/) to ensure the safety of the selected target protein for vaccine development [25]. BLASTp analysis was used to assess sequence similarity with homologous proteins across different strains.

The retrieved sequences were subjected to multiple sequence alignment (MSA) using BioEdit software, employing the built-in ClustalW algorithm to ensure accurate alignment of conserved and variable regions. The resulting alignment was used to evaluate residue-level conservation across YFV strains. To quantitatively assess sequence conservation, Shannon entropy (Hx) analysis was performed using BioEdit. Entropy values were calculated for each aligned amino acid position, where lower entropy scores (Hx<0.5) indicated highly conserved residues, while higher values reflected increased sequence variability. This conservation analysis facilitated the identification of stable and evolutionarily conserved regions within the envelope glycoprotein, supporting its suitability as a rational vaccine target [26].

### Epitope prediction

B-cell epitopes play a crucial role in humoral immunity by inducing antibody responses. The Bepipred Linear Epitope Prediction 2.0 method from the IEDB server (http://tools.iedb.org/bcell/) was used to identify potential B-cell epitopes [27]. MHC-I binding epitopes were identified using the Artificial Neural Network (ANN) 4.0 method from the IEDB server (http://tools.iedb.org/mhci/) [28]. This method predicts epitopes that can bind to human leukocyte antigen (HLA) class I molecules, which are crucial for cytotoxic T-cell responses. MHC-II binding epitopes were predicted using the NN-align 2.3 (NetMHCII 2.3) tool from IEDB (http://tools.iedb.org/mhcii/) [29]. These epitopes stimulate helper T-cells (CD4+), enhancing adaptive immunity by assisting B-cells and cytotoxic T-cells. Before selection, the epitopes were screened based on their antigenicity using VaxiJen v2.0. The default cutoff in VaxiJen for viral proteins is 0.4; however, a more stringent cutoff of 0.5 was applied to enhance specificity and select only highly antigenic candidates. Allergenicity was assessed using AllerTOP v2.1, and toxicity was evaluated using ToxinPred (https://webs.iiitd.edu.in/oscadd/toxipred/supple.php) to identify antigenic, non-allergenic, and non-toxic epitopes suitable for vaccine development [30], 31].

### Epitope conservancy analysis

The conservancy of selected epitopes across various YFV strains was analyzed using the Epitope Conservancy Analysis tool (http://tools.iedb.org/conservancy/). The predicted epitopes were compared with protein sequences from different YFV strains obtained from the NCBI database. The analysis employed a sequence identity threshold of ≥100 % to ensure that only fully conserved epitopes were selected [32].

### Population coverage analysis

To determine the global applicability of the selected epitopes, population coverage analysis was performed using the IEDB Population Coverage Tool (http://tools.iedb.org/population/). This tool estimates HLA allele frequency distribution across different ethnic groups and geographical regions to evaluate immune coverage. The selected MHC-I and MHC-II epitopes were analyzed against global HLA allele frequency databases to estimate their coverage across human populations [33].

### Epitope–HLA interaction analysis

To evaluate binding interactions between epitopes and HLAs, the tertiary structures of selected epitopes were modeled using the PEP-FOLD3 server (https://bioserv.rpbs.univ-paris-diderot.fr/services/PEP-FOLD3/), which provides reliable *de novo* 3D structure predictions for short peptides [34]. For MHC class I analysis, docking was performed with HLA-A*02:01 (PDB ID: 1QEW) and HLA-A*11:01 (PDB ID: 5WJL). Similarly, MHC class II peptides were docked with HLA-DRB101:01 (PDB ID: 2G9H) *and *HLA-DRB1*01:01 (PDB ID: 1BX2). Docking analysis was carried out using the ClusPro 2.0 server (https://cluspro.bu.edu/login.php), which clusters docked conformations based on energy scores and interface complementarity [35]. Interaction analysis was further performed using LigPlot+ v2.2.9 Discovery Studio Visualizer to visualize hydrogen bonds and hydrophobic interactions between peptides and HLA molecules.

### Vaccine construct design

The multi-epitope vaccine construct was assembled by joining B-cell and T-cell epitopes using appropriate linkers. An adjuvant (beta-defensin-3) was added at the N-terminus to promote the innate immune response [36]. The GPGPG linker was used for MHC-II epitopes, preventing junctional immunodominance and ensuring effective recognition by CD4^+^ T-cells [37]. The AAY linker was used for MHC-I epitopes to enhance proteasomal processing and facilitate antigen presentation [38]. The KK linker was utilized for B-cell epitopes to maintain structural integrity and stability [39]. Moreover, a PADRE sequence (AKFVAAWTLKAAA) was added to enhance T-cell response and immunogenicity. Finally, a 6× histidine tag was placed at the C-terminus for purification and identification in the host expression system. The antigenicity of the final vaccine construct was evaluated using the VaxiJen v2.0 server, and allergenicity was assessed using the AllerTOP v2.1 server [40].

### Physicochemical properties analysis

The physicochemical properties of the vaccine construct were analyzed using the ExPASy ProtParam tool (https://web.expasy.org/protparam/), including molecular weight, theoretical isoelectric point (pI), instability index, aliphatic index, and GRAVY score [41], 42]. Protein solubility was predicted using the SoluProt server v1.0 (https://loschmidt.chemi.muni.cz/soluprot/), to evaluate expression potential in *Escherichia coli* [43].

### Secondary structure prediction

The PSIPRED server (http://bioinf.cs.ucl.ac.uk/psipred/) was used to predict the secondary structure of the vaccine construct. This tool utilizes a neural network to analyze the protein sequence and predict structural components such as alpha-helices, beta-sheets, and random coils [44], 45].

### Tertiary structure prediction and validation

The tertiary structure of the designed vaccine was predicted using AlphaFold3 (https://alphafoldserver.com/), a deep-learning tool that accurately predicts protein structures. It predicts structures using evolutionary history, physical constraints, and deep learning, yielding reliable results [46]. To improve the structure in terms of side-chain packing, overall stability, and structural accuracy, the Galaxy Refine server (https://galaxy.seoklab.org/cgi-bin/submit.cgi?type=REFINE) was used to refine the predicted tertiary structure of the vaccine [47]. The refined structure was validated through Ramachandran (RC) plot analysis using the PROCHECK server (https://saves.mbi.ucla.edu/) to evaluate backbone dihedral angles for proper folding [48]. Moreover, the overall quality and reliability of the refined structure were assessed using the ERRAT program [49], 50]. In addition, the refined tertiary structure was evaluated using the ProSA-web server (https://prosa.services.came.sbg.ac.at/prosa.php), which provides both global and local quality assessments based on statistical potentials derived from experimentally resolved protein structures in the Protein Data Bank (PDB). ProSA generates a Z-score to assess overall model quality relative to native proteins of similar size and a residue-wise energy profile to identify potential local structural anomalies [51]. Furthermore, structural quality was independently assessed using the QMEANDisCo scoring function implemented in the SWISS-MODEL Structure Assessment tool (https://swissmodel.expasy.org/assess) to evaluate the global and local reliability of the predicted vaccine construct [52].

### Prediction of discontinuous B-cell epitopes

Discontinuous B-cell epitopes were predicted using the ElliPro server (http://tools.iedb.org/ellipro/) available at the Immune Epitope Database (IEDB). The vaccine structure was submitted to the server, and epitope prediction was performed under default parameters (the minimum score was set to 0.5, and the residue clustering distance was 6 Å). For the prediction of discontinuous epitopes, ElliPro utilizes a geometric approximation of protein shape and computes a protrusion index (PI) score for each predicted epitope. Predicted epitopes were analyzed based on their PI scores, which were ranked in descending order, indicating greater epitope potential [53].

### Disulfide engineering

Disulfide engineering was performed on the designed vaccine construct using the Disulfide by Design 2 (DbD2) tool (http://cptweb.cpt.wayne.edu/DbD2/) to enhance structural stability. The tertiary structure of the vaccine construct was uploaded to the DbD2 server, and disulfide bonds were predicted using default parameters except for the χ^3^ angle, which was set to −87° or +97° ± 30°, and the Cα–Cβ–Sγ angle, which was set to 114.6° ± 10°. The tool identified potential residue pairs suitable for disulfide bond formation, and candidate mutations were selected based on geometric constraints and structural stability scores [54], 55].

### Molecular docking and interaction analysis

Molecular docking of the designed vaccine construct with the human innate immune receptors TLR2 and TLR8 was performed using the ClusPro 2.0 server (https://cluspro.bu.edu/login.php). Docking was carried out using a blind docking strategy, in which no predefined binding sites were specified and the entire receptor surface was explored to avoid bias in complex formation. ClusPro 2.0 employs a rigid-body docking approach based on Fast Fourier Transform (FFT) algorithms to generate and rank protein–protein interaction conformations [35]. The docking procedure involved energy-based scoring and clustering of docked poses, and the most representative clusters with the lowest weighted interaction scores and highest population density were selected for further analysis. The docking scores reported by ClusPro represent weighted interaction scores (arbitrary units) and do not correspond to absolute binding free energies.

The intermolecular interactions within the selected docked complexes were analyzed using PDBsum (https://www.ebi.ac.uk/thornton-srv/software/PDBsum1/), a structural bioinformatics tool that provides detailed protein–protein interaction profiles [56]. PDBsum was used to identify and characterize hydrogen bonds, salt bridges, and hydrophobic interactions stabilizing the vaccine–receptor complexes.

In addition, CoCoMaps2 (https://aocdweb.com/BioTools/cocomaps2/) was employed to further analyze the non-covalent interactions and interface properties of the docked complexes [57]. CoCoMaps2 was used to evaluate the atomic interaction distribution and calculate the buried surface area (BSA) at the vaccine–receptor interface, providing complementary insights into interface stability and interaction composition.

### Molecular dynamics simulations

Molecular dynamics (MD) simulations of the vaccine–receptor complexes were performed using the AMBER software suite to evaluate their dynamic behavior and stability, employing the ff19SB force field. A TIP3P water model was utilized for solvation and placed in a 12 Å simulation box to ensure sufficient hydration. Furthermore, sodium and chloride ions were included to neutralize the system and maintain physiological conditions [58]. To remove unwanted steric clashes and unfavorable interactions, a two-step energy minimization of 20,000 steps was performed. The system was then equilibrated for five nanoseconds (ns) at a constant temperature of 298 K and pressure of 1 bar. To ensure the reliability and reproducibility of the results, three independent MD simulation replicas were performed, each subjected to identical equilibration and production conditions. The final production phase MD simulations were carried out for 100 ns under the same conditions, with 10,000 trajectory frames collected at intervals of 10 ps. The flexibility and binding interactions of the vaccine were analyzed using multiple parameters. Root mean square deviation (RMSD), root mean square fluctuation (RMSF), and radius of gyration (Rg) were calculated to assess structural stability and compactness. Principal component analysis (PCA) was performed to evaluate conformational dynamics. In addition, hydrogen bond analysis was carried out to examine the stability and interaction patterns between the vaccine construct and the TLR2 receptor throughout the simulation [59]. Hydrogen bond analysis was performed using the CPPTRAJ module of the AMBER software suite. Both total hydrogen bonds, including intra- and intermolecular interactions within the entire complex, and intermolecular hydrogen bonds between the vaccine construct and the TLR2 receptor were evaluated. Hydrogen bonds were identified based on standard geometric criteria, using a heavy atom (donor–acceptor) distance cutoff of ≤3.5 Å and a donor–hydrogen–acceptor angle cutoff of ≥120°. The number of hydrogen bonds was calculated for each trajectory frame throughout the simulation to assess interaction stability and persistence.

### Immune simulation

To analyze the immune response of the designed multi-epitope vaccine, the C-ImmSim server **(** https://kraken.iac.rm.cnr.it/C-IMMSIM/) was used, which simulates the human immune response based on machine learning algorithms. The simulation was carried out with three different doses using default parameters, including a random seed of 12345, a simulation volume of 100, and 300 simulation steps, to assess the vaccine’s capability to induce B-cell and T-cell responses, cytokine release, and immunological memory formation. The injection included 1000 antigenic molecules and an adjuvant concentration of 100 arbitrary units [60]. This assessment also measured B-cell activation, antibody secretion, and memory B-cell generation, ensuring a sustained defensive response. Furthermore, CD4+ helper T-cell and CD8+ cytotoxic T-cell responses were analyzed to assess immune defense at the cellular level. Cytokine and interleukin profiles were analyzed to determine the immunological activity of the vaccine [61].

### *In silico* cloning

The vaccine construct was reverse-translated into a nucleotide sequence using the EMBOSS Backtranseq tool (https://www.ebi.ac.uk/jdispatcher/st/emboss_backtranseq) [62]. To improve expression in the host system *E. coli* strain K12, the codon sequence was optimized using the ExpOptimizer tool (https://www.novoprolabs.com/tools/codon-optimization) to match the preferred codon usage of *E. coli strain K12*. The modified vaccine sequence was inserted into the pBR322 expression vector for recombinant expression using SnapGene software, which confirmed the proper insertion of the gene and its suitability for expression in the selected bacterial system [63].

### Research ethics

Not applicable.

### Informed consent

Not applicable.

## Results

### Protein selection and sequence retrieval

The envelope glycoprotein was selected as the target antigen for the development of an *in silico* multi-epitope vaccine against yellow fever virus. The amino acid sequence of the envelope glycoprotein, consisting of 481 residues, was retrieved in FASTA format from the UniProt database under the accession number D0VF74. Antigenicity prediction using VaxiJen yielded a score of 0.5457, indicating good antigenic potential. In addition, allergenicity assessment performed using AllerTOP classified the protein as non-allergenic, supporting its suitability as a vaccine candidate.

**Figure 2:**
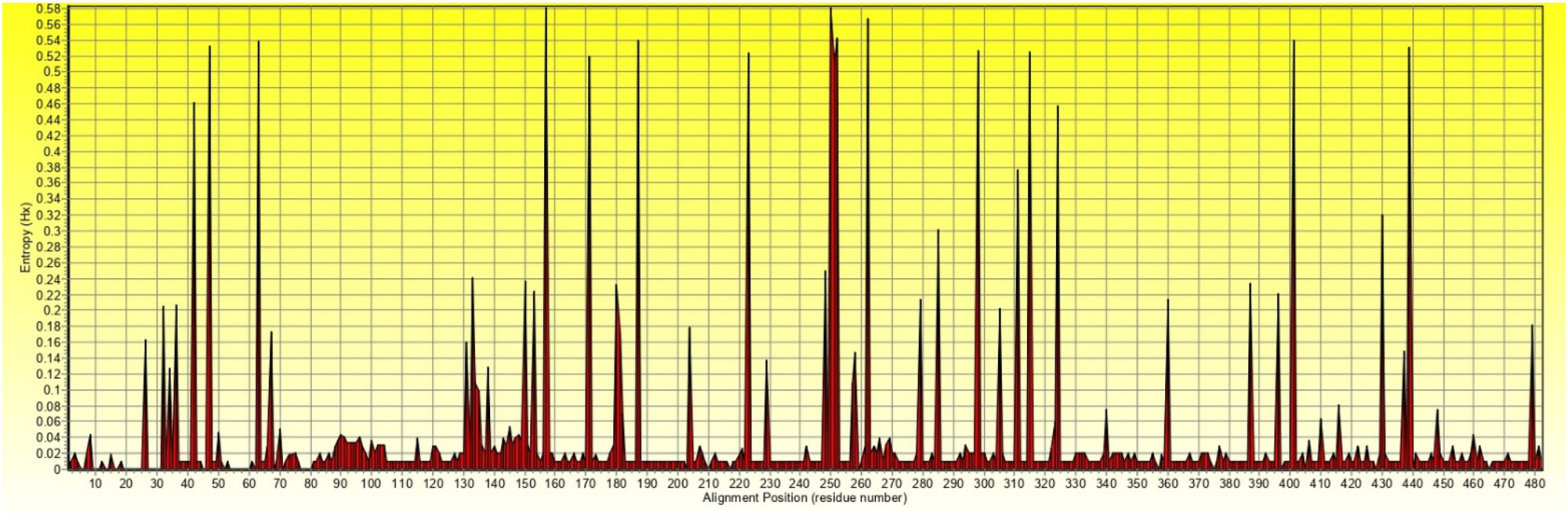
Shannon entropy (Hx) plot showing residue-wise sequence conservation of the yellow fever virus envelope glycoprotein based on multiple sequence alignment of 751 homologous sequences retrieved through BLASTp analysis. Lower entropy values indicate highly conserved residues, whereas higher values represent variable regions.

To evaluate sequence conservation across YFV strains, BLASTp analysis was performed against the NCBI non-redundant protein database. A total of 751 homologous envelope glycoprotein sequences, each exhibiting 100 % query coverage and greater than 90 % sequence identity, were selected for downstream analysis. These sequences were subjected to multiple sequence alignment using BioEdit, and residue-level conservation was quantitatively assessed using Shannon entropy analysis. The Shannon entropy plot demonstrated that the majority of amino acid positions displayed low entropy values (Hx<0.5), indicating a high degree of sequence conservation among the analyzed YFV strains (Figure 2). Only a small number of residues exhibited higher entropy values, reflecting localized sequence variability. The predominance of conserved regions highlights the evolutionary stability of the envelope glycoprotein and supports its selection as a reliable and robust target for multi-epitope vaccine design.

### Epitope prediction

The IEDB tool was used to predict B-cell and T-cell epitopes. Only epitopes that were non-allergenic, non-toxic, had an IC50 value below 100, and a VaxiJen score above 0.5 were selected for the vaccine construct to ensure effective activation of helper T-cells and promote a balanced immune response. Based on these parameters, six potential epitopes were selected for vaccine construct design, including two epitopes from MHC-I, two from MHC-II, and two B-cell epitopes. The selected B-cell linear epitopes are given in Table 1, MHC-I epitopes are detailed in Table 2, and MHC-II epitopes are presented in Table 3.

**Table 1:** Selected B-cell epitopes of the target protein.

Protein name	Epitope	Antigenicity	Allergenicity	Toxicity
Envelope glycoprotein	LSGSQEAEFTG	0.8263	Non-allergen	Non-toxic
	AWDFSSAGG	1.1618	Non-allergen	Non-toxic

**Table 2:** Selected MHC-I epitopes of the target protein.

Protein name	Epitope	Antigenicity	Allergenicity	Toxicity
Envelope glycoprotein	RVKLSALTLK	1.4407	Non-allergen	Non-toxic
	VIMMFLSLGV	1.0222	Non-allergen	Non-toxic

**Table 3:** Selected MHC-II epitopes of the target protein.

Protein name	Epitope	Antigenicity	Allergenicity	Toxicity
Envelope glycoprotein	KIQYVIRAQLHVGAK	1.0370	Non-allergen	Non-toxic
	WVGINTRNMTMSMSM	1.5191	Non-allergen	Non-toxic

### Epitope conservancy analysis

The epitope conservancy analysis of the yellow fever virus envelope glycoprotein revealed 100 % sequence conservation across multiple YFV strains, as shown in Table 4. The selected B-cell, MHC-I, and MHC-II epitopes exhibited complete identity across all analyzed sequences, confirming that the selected epitopes are highly conserved and evolutionarily preserved, making them strong candidates for vaccine construction. The high degree of epitope conservation may contribute to a broad cross-protective immune response against various strains of YFV, thereby increasing vaccine efficacy and providing long-lasting protection.

**Table 4:** List of selected epitopes with their percentage of conservancy among yellow fever virus.

Epitopes	B-cell		MHC I		MHC II	
Accession No.	LSGSQEAEFTG	AWDFSSAGG	RVKLSALTLK	VIMMFLSLGV	KIQYVIRAQLHVGAK	WVGINTRNMTMSMSM
QUW16862.1	100 %	100 %	100 %	100 %	100 %	100 %
AXY92189.1	100 %	100 %	100 %	100 %	100 %	100 %
QUW16873.1	100 %	100 %	100 %	100 %	100 %	100 %
AFU76906.1	100 %	100 %	100 %	100 %	100 %	100 %
AXY92186.1	100 %	100 %	100 %	100 %	100 %	100 %
AVT50844.1	100 %	100 %	100 %	100 %	100 %	100 %
AXY92187.1	100 %	100 %	100 %	100 %	100 %	100 %
URT56408.1	100 %	100 %	100 %	100 %	100 %	100 %
ACY40742.1	100 %	100 %	100 %	100 %	100 %	100 %
WGJ78830.1	100 %	100 %	100 %	100 %	100 %	100 %
ACY40729.1	100 %	100 %	100 %	100 %	100 %	100 %
AMZ00440.1	100 %	100 %	100 %	100 %	100 %	100 %
ACY40711.1	100 %	100 %	100 %	100 %	100 %	100 %
ACY40712.1	100 %	100 %	100 %	100 %	100 %	100 %
QZW25499.1	100 %	100 %	100 %	100 %	100 %	100 %
QEF75576.1	100 %	100 %	100 %	100 %	100 %	100 %
QZW25505.1	100 %	100 %	100 %	100 %	100 %	100 %
QZW25512.1	100 %	100 %	100 %	100 %	100 %	100 %
WGJ78829.1	100 %	100 %	100 %	100 %	100 %	100 %
QZW25504.1	100 %	100 %	100 %	100 %	100 %	100 %

### Population coverage analysis

**Figure 3:**
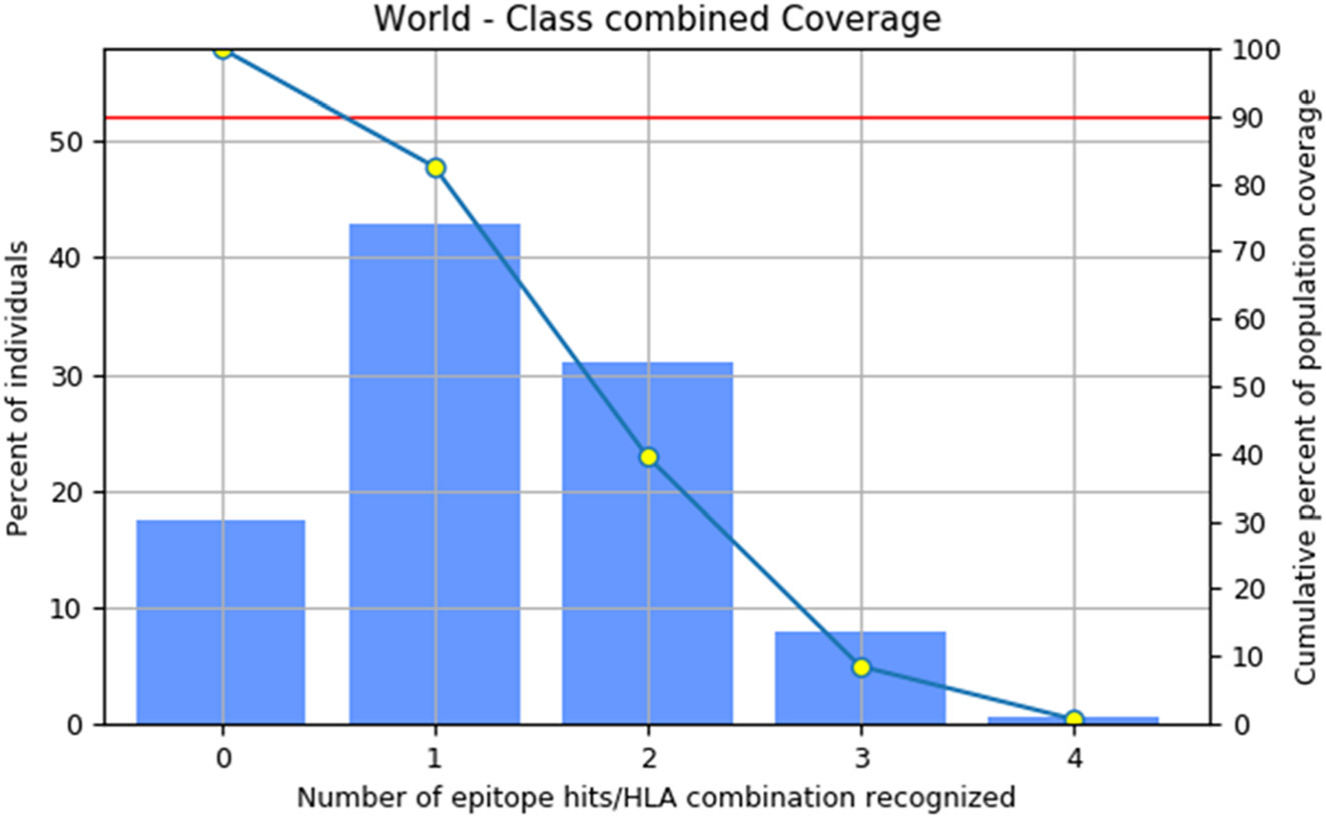
Global population coverage of combined MHC-I and MHC-II epitopes.

**Figure 4:**
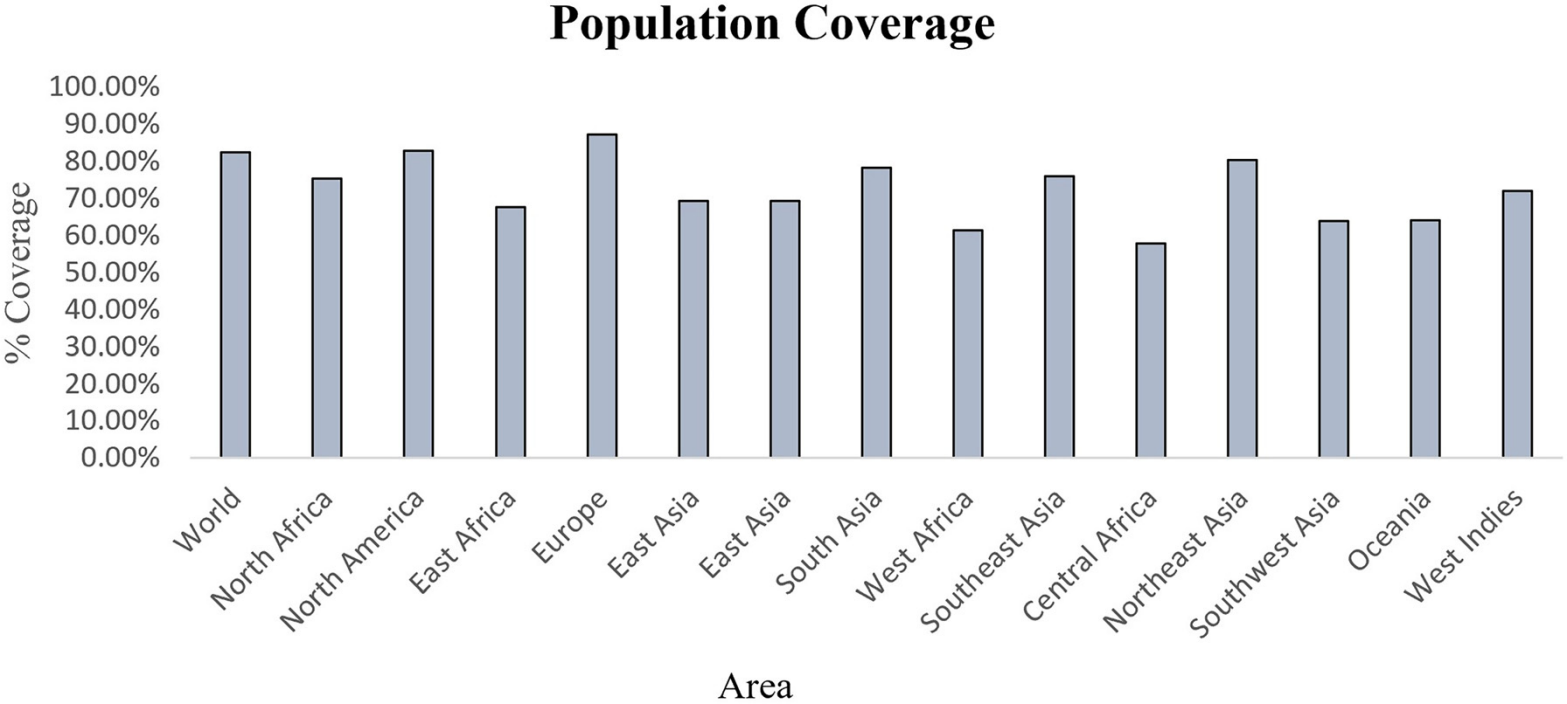
Regional population coverage of combined MHC-I and MHC-II epitopes.

The combined population coverage analysis of MHC-I and MHC-II epitopes showed global applicability, ensuring that the vaccine construct would be effective across various ethnic groups. The worldwide population coverage was estimated to be above 83.03 %, as shown in Figure 3, which correlates with the strong immunogenic potential of the selected epitopes. In specific regions, Europe exhibited the highest population coverage (87.42 %), followed by North America (83.03 %) and Northeast Asia (80.37 %). South Asia (78.32 %), Southeast Asia (76.03 %), and North Africa (75.42 %) also demonstrated substantial coverage, indicating the broad applicability of the selected epitopes across diverse populations. East Asia (69.42 %), Oceania (64.24 %), and Southwest Asia (63.90 %) showed moderate coverage, whereas West Africa (61.35 %) and Central Africa (57.90 %) exhibited comparatively lower coverage, as shown in Figure 4, indicating that further refinement of epitopes could improve inclusivity. These results confirm that the selected MHC-I and MHC-II epitopes provide broad population coverage, making the vaccine construct a strong candidate for immunization against yellow fever virus.

### Epitope–HLA interaction analysis

**Figure 5:**
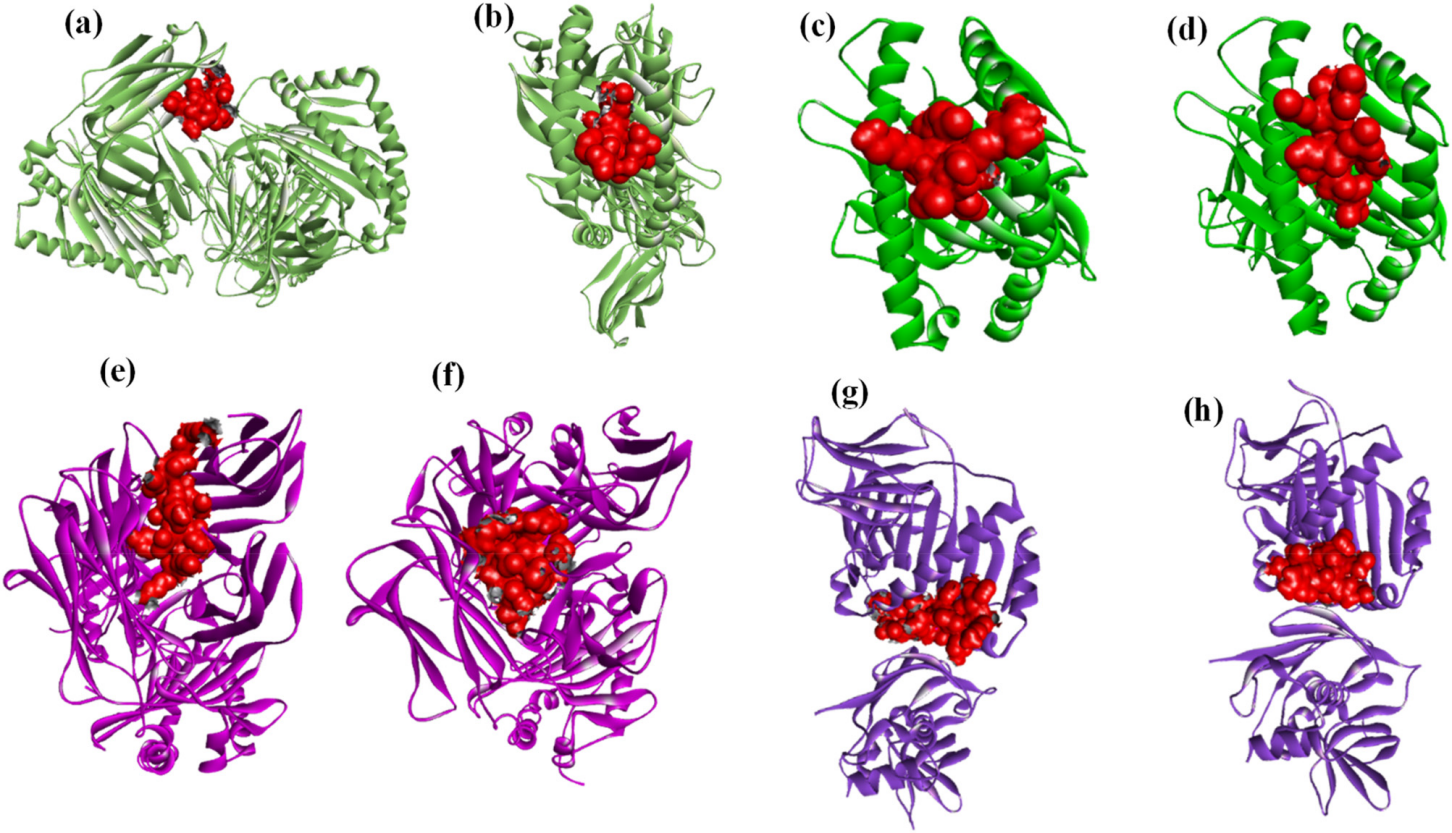
Molecular docking visualization of selected epitopes bound to MHC molecules. (a) Epitope 1 with HLA-A*11:01, (b) epitope 2 with HLA-A*11:01, (c) epitope 1 with HLA-A*02:01, (d) epitope 2 with HLA-A*02:01, (e) epitope 3 with HLA-DRB1*01:01, (f) epitope 4 with HLA-DRB1*01:01, (g) epitope 3 with HLA-DRB1*15:01, (h) epitope 4 with HLA-DRB1*15:01. Red spheres represent the docked epitope peptides at the binding groove of MHC molecules visualized using Discovery Studio Visualizer.

Molecular docking analysis was carried out using ClusPro 2.0 to estimate the binding interactions between the selected epitopes and MHC-I and MHC-II molecules. The docked complexes were visualized using Discovery Studio Visualizer, as shown in Figure 5, and hydrogen bonding interactions were evaluated using LigPlot+ v2.2.9. Both HLA-A*11:01 and HLA-A*02:01 demonstrated stable binding with Epitope 1 (RVKLSALTLK), forming 11 and 7 hydrogen bonds, respectively, with docking scores of −547.4 and −655.2. Similarly, HLA-A*11:01 and HLA-A*02:01 showed stable binding with Epitope 2 (VIMMFLSLGV), with docking scores of −852.0 and −953.5, forming 3 and 4 hydrogen bonds, respectively. Regarding MHC-II, Epitope 3 (KIQYVIRAQLHVGAK) interacted with HLA-DRB1*01:01* and *HLA-DRB1*15:01, exhibiting docking scores of −902.1 and −947.8, respectively, while forming 13 and 8 hydrogen bonds. Epitope 4 (WVGINTRNMTMSMSM) yielded the most negative docking scores of −915.8 and −973.4 with HHLA-DRB1*01:01 and HLA-DRB1*15:01, forming 9 and 8 hydrogen bonds, respectively, as presented in Table 5. The favorable docking scores, along with significant hydrogen bonding, suggest strong and stable interactions, validating the selected epitopes’ capacity for efficient antigen presentation and T-cell stimulation, thereby supporting their inclusion in multi-epitope vaccine design.

**Table 5:** Molecular docking results of selected T-cell epitopes with their respective MHC class I and II molecules.

Epitopes	Epitope class	Sequences	Receptors	Docking score	Members	Hydrogen bonds
Epitope 1	MHC-I	RVKLSALTLK	HLA-A*11:01	−547.4	233	11
Epitope 1	MHC-I	RVKLSALTLK	HLA-A*02:01	−655.2	220	7
Epitope 2	MHC-I	VIMMFLSLGV	HLA-A*11:01	−852.0	279	3
Epitope 2	MHC-I	VIMMFLSLGV	HLA-A*02:01	−953.5	301	4
Epitope 3	MHC-I I	KIQYVIRAQLHVGAK	HLA-DRB1*01:01	−902.1	584	13
Epitope 3	MHC-I I	KIQYVIRAQLHVGAK	HLA-DRB1*15:011	−947.8	283	8
Epitope 4	MHC-I I	WVGINTRNMTMSMSM	HLA-DRB1*01:01	−915.8	527	9
Epitope 4	MHC-I I	WVGINTRNMTMSMSM	HLA-DRB1*15:011	−973.4	426	8

### Vaccine construct design

**Figure 6:**
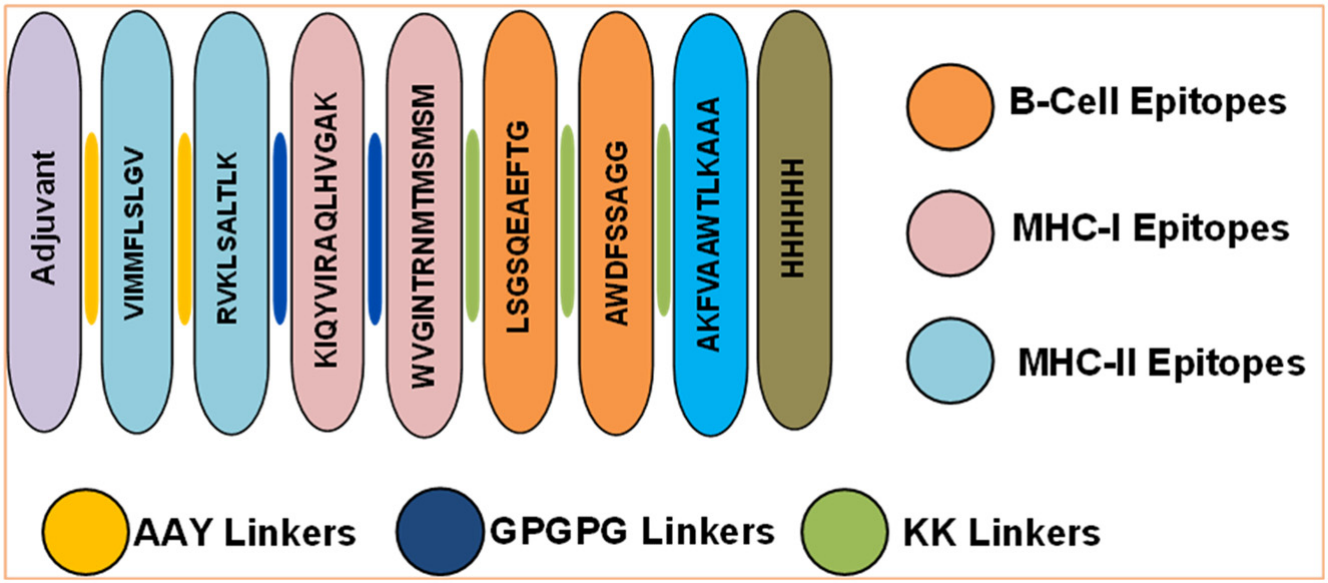
The arrangement of adjuvant, linkers and epitopes in candidate vaccine construct.

The designed vaccine construct, as shown in Figure 6, consists of an adjuvant (beta-defensin-3) linked to MHC-I epitopes using the AAY linker. MHC-II epitopes were linked using GPGPG linkers, while B-cell epitopes were linked using KK linkers. The final vaccine construct consisted of 156 amino acids and was predicted to be non-allergenic, and had a VaxiJen score of 0.7946, as evaluated using AllerTOP, and VaxiJen v2.0, respectively.

### Physicochemical properties analysis

The physicochemical analysis indicated that the designed vaccine construct has a molecular weight of 17,059.18  Da with a basic nature, as reflected by a theoretical pI of 10.36. It contains 156 amino acids, including 5 negatively charged (Asp + Glu) and 28 positively charged (Arg + Lys) residues. The estimated half-life was more than 10 h in *E. coli* and approximately 30 h in mammalian reticulocytes, suggesting potential for sustained expression. An instability index (II) of 20.79 classified the construct as stable, while an aliphatic index of 72.63 indicates possible thermal stability. The GRAVY score of −0.263 suggests a hydrophilic nature. The construct also showed a solubility score of 0.793 in *E. coli*, as presented in Table 6. Overall, these properties indicate that the construct possesses characteristics generally associated with soluble and stable proteins.

**Table 6:** Physicochemical properties of designed vaccine.

Physicochemical properties	Results
Number of amino acids	156
Molecular weight	17,059.18
Theoretical pI	10.36
Total negatively charged residues (Asp + Glu)	5
Total positively charged residues (Arg + Lys)	28
Total number of atoms	2,419
Estimated half-life (mammalian reticulocytes, *in vitro*)	30 h
Estimated half-life (*Escherichia coli*, *in vivo*)	>10 h
Instability index (II)	20.79
Aliphatic index	72.63
Grand average of hydropathicity (GRAVY)	−0.263
Solubility	0.793

**Table 7:** Amino acid residues of discontinuous epitopes with their scores.

No.	Residues	Length	Score
1	A:G1, A:I2, A:I3, A:N4, A:T5, A:L6, A:Q7, A:K8, A:Y9, A:Y10, A:C11, A:R12, A:V13, A:R14, A:G15, A:G16, A:A19, A:V20, A:L21, A:S22, A:C23, A:Q29, A:I30, A:G31, A:K32, A:C33, A:S34, A:T35, A:R36, A:G37, A:R38, A:K39	32	0.739
2	A:A90, A:K91, A:G92, A:P93, A:G94, A:W97, A:V98	7	0.717
3	A:F122, A:T123, A:G124, A:K125, A:K126, A:W128, A:D129, A:S131, A:S132, A:A133, A:G134, A:G135, A:K136	13	0.668
4	A:S108, A:H152, A:H153, A:H156	4	0.639
5	A:R62, A:V63, A:S66, A:A67, A:T69, A:L70, A:K71, A:G72, A:P73, A:G74, A:P75, A:M111, A:L114, A:S115, A:G116, A:S117, A:Q118, A:E119, A:A120, A:E121	20	0.598
6	A:I100, A:N101, A:T102, A:R103, A:N104, A:M105, A:T106	7	0.596

### Secondary structure prediction

**Figure 7:**
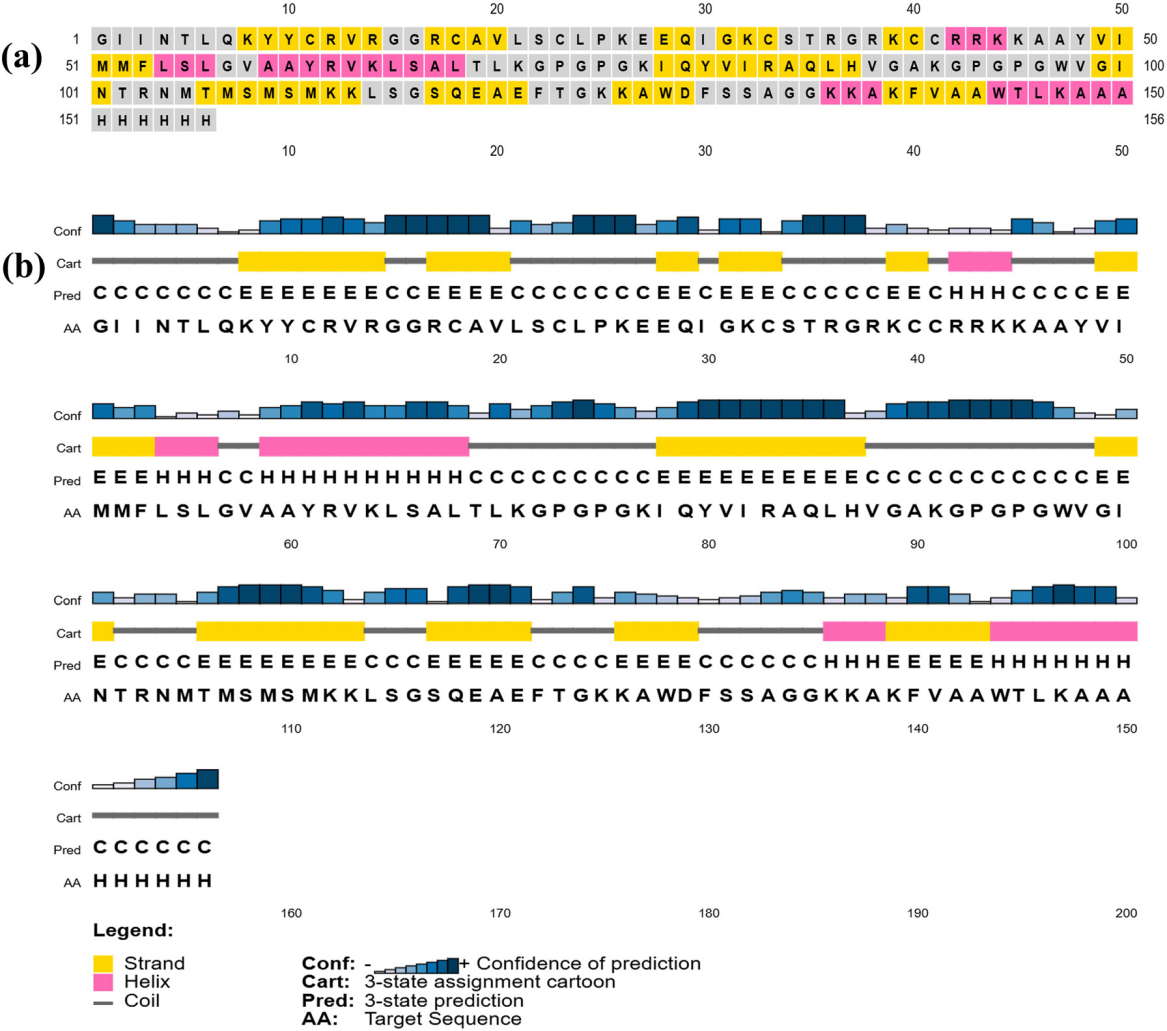
The secondary structure configuration of the vaccine candidate. (a) Graphical representation of secondary structure of vaccine construct predicted by PSIPRED server. (b) Demonstration of amino acid units encoding for secondary units Helix, sheets, and coil with their prediction scores.

The secondary structure of the designed vaccine construct was predicted using the PSIPRED tool, as illustrated in Figure 7. The analysis showed that the protein structure is well organized into clearly defined alpha-helices, beta-strands, and coils, which may contribute to the functional properties of the vaccine. The results indicated that 29 (18.6 %) amino acids provide structural flexibility and stability as alpha-helices (pink regions). Beta-strands (yellow regions) are formed by 58 (37.2 %) amino acids that provide rigidity to the protein framework. The remaining 69 (44.2 %) amino acids that form coils (grey regions) are crucial for protein folding and interactions. Such structural composition suggests that the vaccine construct maintains a balanced secondary structure, which may support proper folding, receptor binding, and immunogenic response.

### Tertiary structure prediction and validation

**Figure 8:**
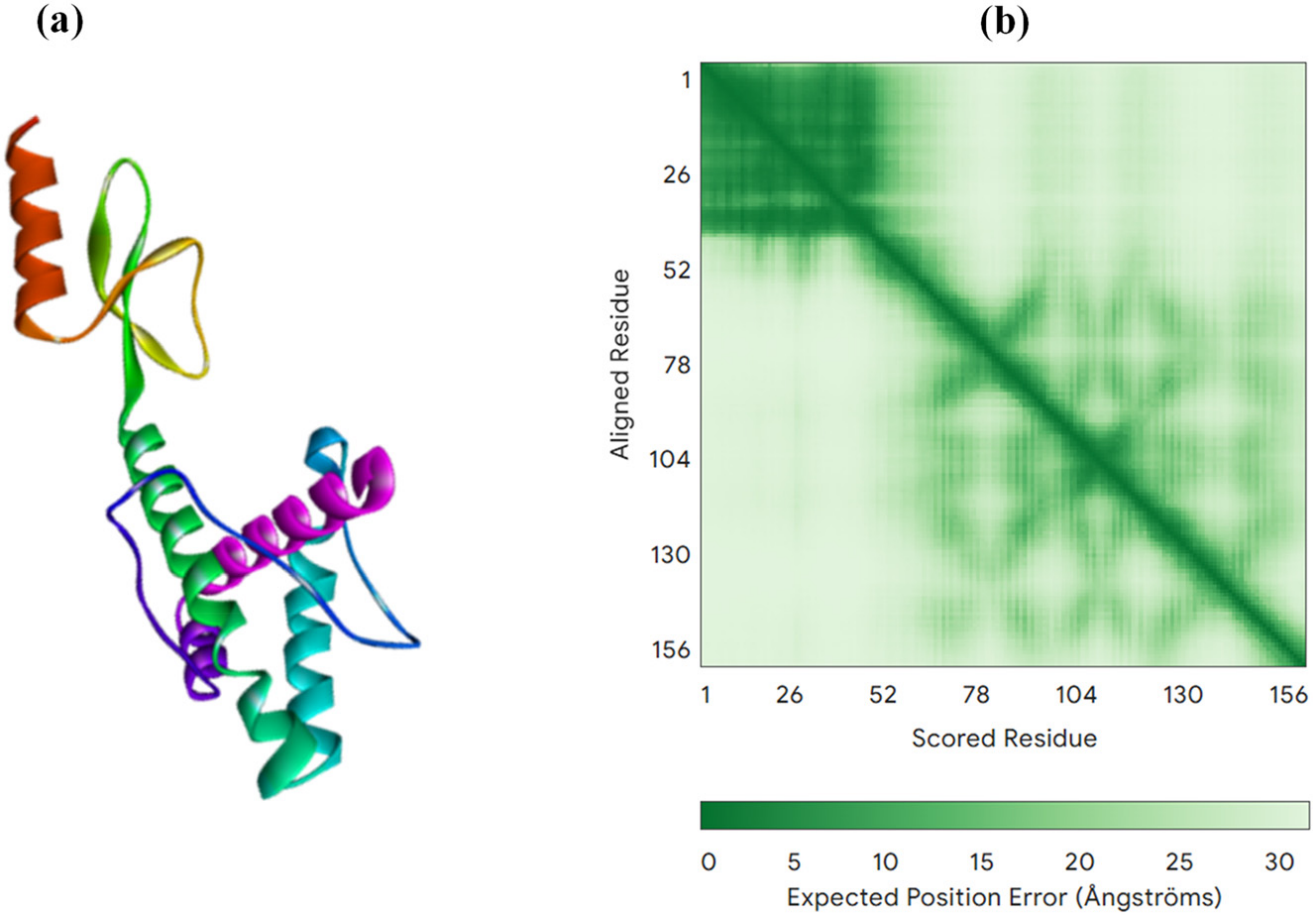
Predicted tertiary structure of the vaccine construct using AlphaFold3. (a) Three-dimensional structure shown in ribbon representation. (b) Predicted aligned error (PAE) plot illustrating residue-wise positional confidence.

The three-dimensional structure of the designed multi-epitope vaccine construct was initially predicted using the AlphaFold3 server. The AlphaFold3 prediction produced a predicted TM-score (pTM/ipTM) of 0.27, indicating moderate global confidence, which is expected for artificial, linker-rich multi-epitope vaccine constructs. The predicted tertiary structure of the vaccine construct is shown in Figure 8a. In addition, the predicted aligned error (PAE) plot highlighted higher positional confidence within structured regions and increased flexibility across linker and terminal segments, reflecting the dynamic nature of the construct (Figure 8b).

**Figure 9:**
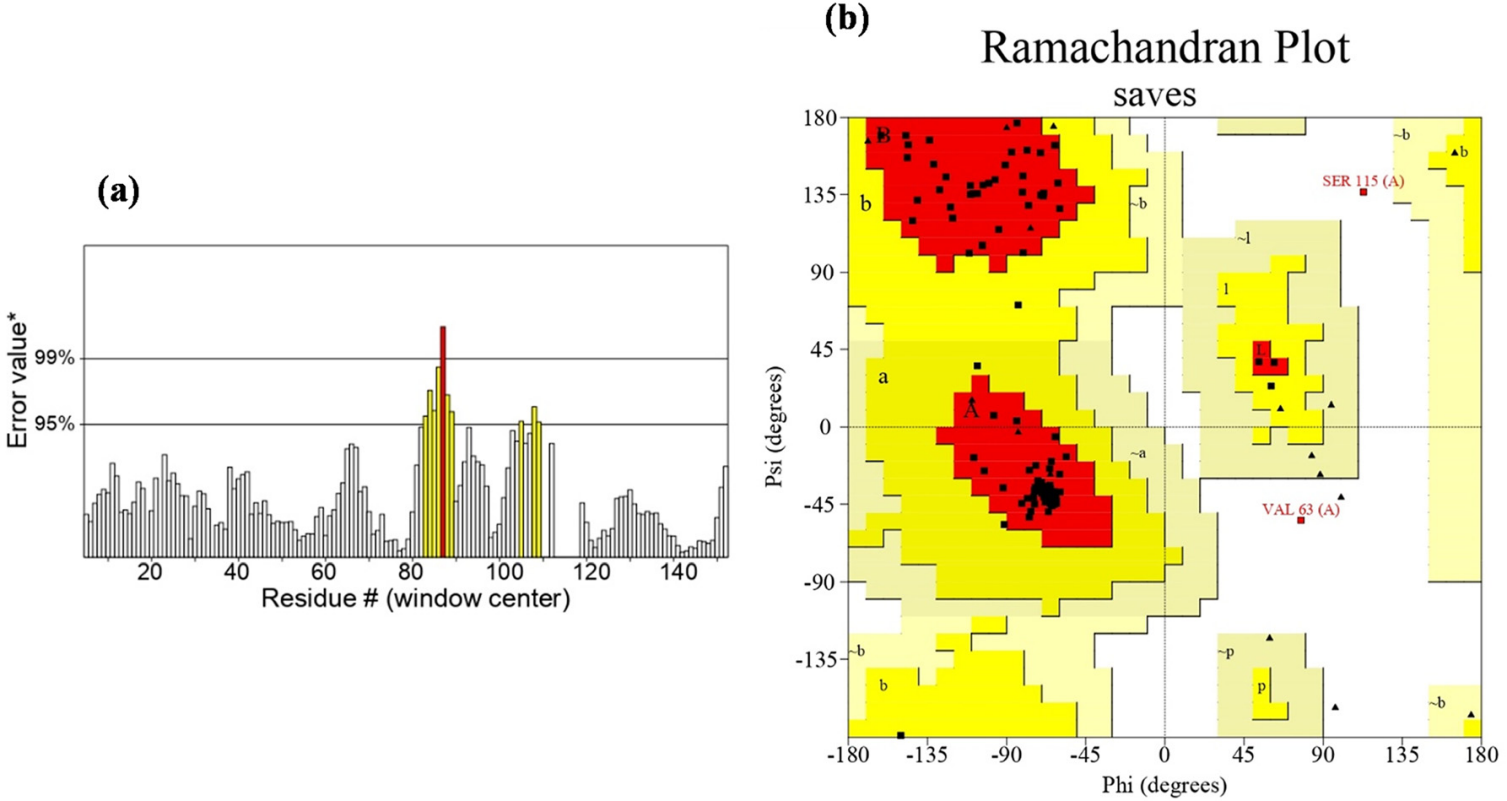
Structural validation of the refined vaccine model. (a) ERRAT quality assessment showing an overall score of 92.7 %. (b) Ramachandran plot indicating residue distribution, with 94.7 % in favored regions.

Following AlphaFold3 prediction, the model was refined using the GalaxyRefine server to improve side-chain packing and overall structural stability prior to validation. Structural validation was then performed using multiple complementary approaches. Ramachandran plot analysis demonstrated that 89.4 % of residues were located in the most favored regions before refinement, which increased to 94.7 % after refinement, indicating improved backbone dihedral angle geometry and stereochemical quality (Figure 9b). Two residues, SER115 and VAL63, were observed in disallowed regions. These residues are positioned within flexible loop or linker regions of the multi-epitope construct and do not constitute part of the structured core, which is considered acceptable for chimeric vaccine designs. The refined structure was further evaluated using the ERRAT server to assess non-bonded atomic interactions. The model achieved an ERRAT quality score of 92.7 %, indicating low levels of structural errors and acceptable overall model quality (Figure 9a).

**Figure 10:**
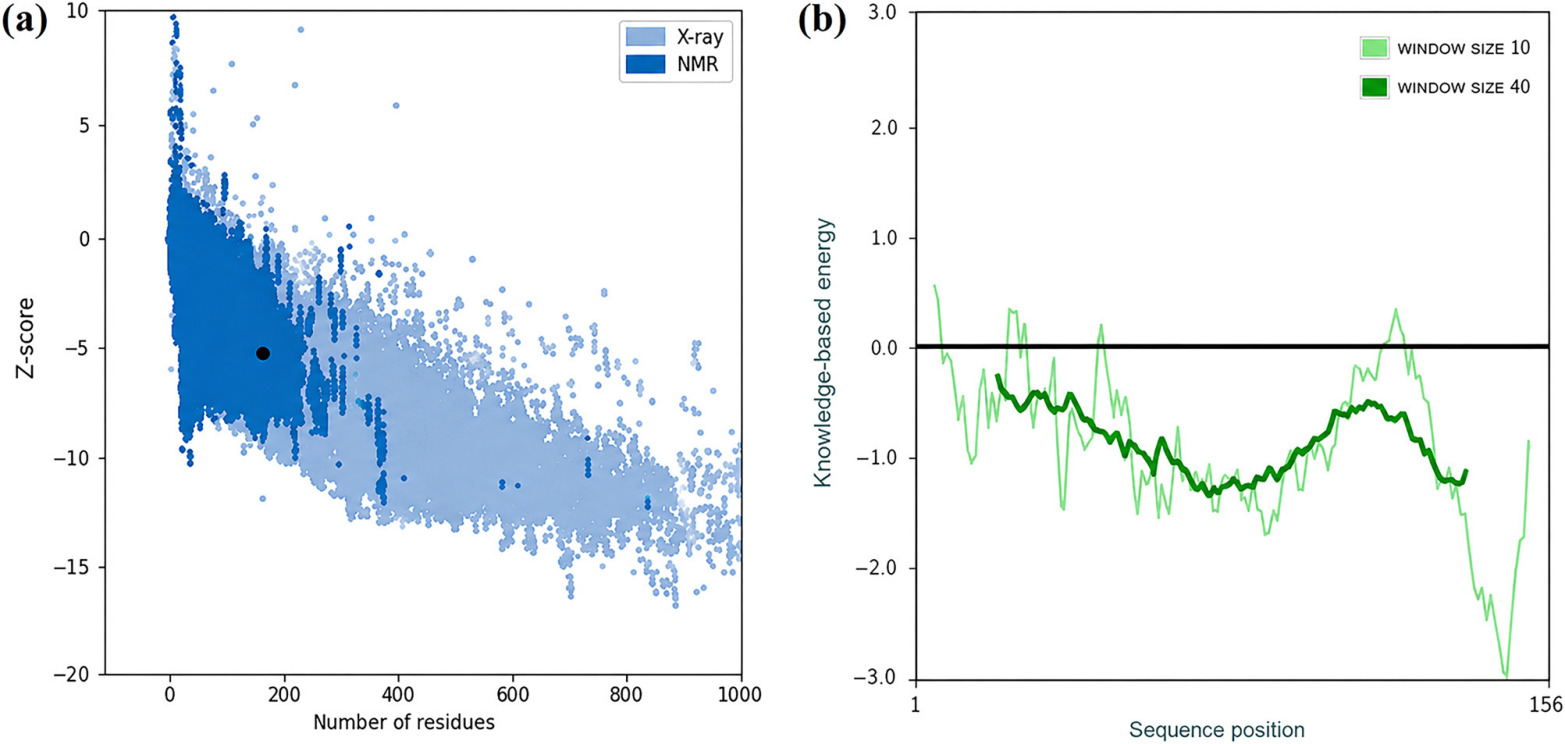
Validation of the predicted tertiary structure of the vaccine using ProSA-web server. (a) Z-score plot showing the overall quality of the model (Z-score: −5.38; indicated by a black dot). (b) Residue-wise energy profile illustrating local structural quality.

To further evaluate the global and local structural quality, the refined vaccine construct was analyzed using the ProSA-web server. The model exhibited a ProSA Z-score of −5.38, which lies within the range of experimentally resolved native protein structures of comparable size (∼156 residues), suggesting an acceptable overall model quality (Figure 10a). Moreover, the knowledge-based energy profile generated by ProSA-web server showed predominantly negative energy values across the sequence, indicating favorable residue environments and the absence of major local structural anomalies (Figure 10b). Finally, structural assessment using the QMEANDisCo scoring function implemented in the SWISS-MODEL Structure Assessment tool yielded a global QMEANDisCo score of 0.41 ± 0.07, which falls within the acceptable range for small, multi-epitope protein constructs.

Overall, the combined results from AlphaFold3 prediction, refinement, and multiple validation tools suggest that the designed multi-epitope vaccine construct possesses acceptable structural quality, stability, and reliability, supporting its suitability for subsequent molecular docking and molecular dynamics analyses.

### Prediction of discontinuous B-cell epitopes

**Figure 11:**
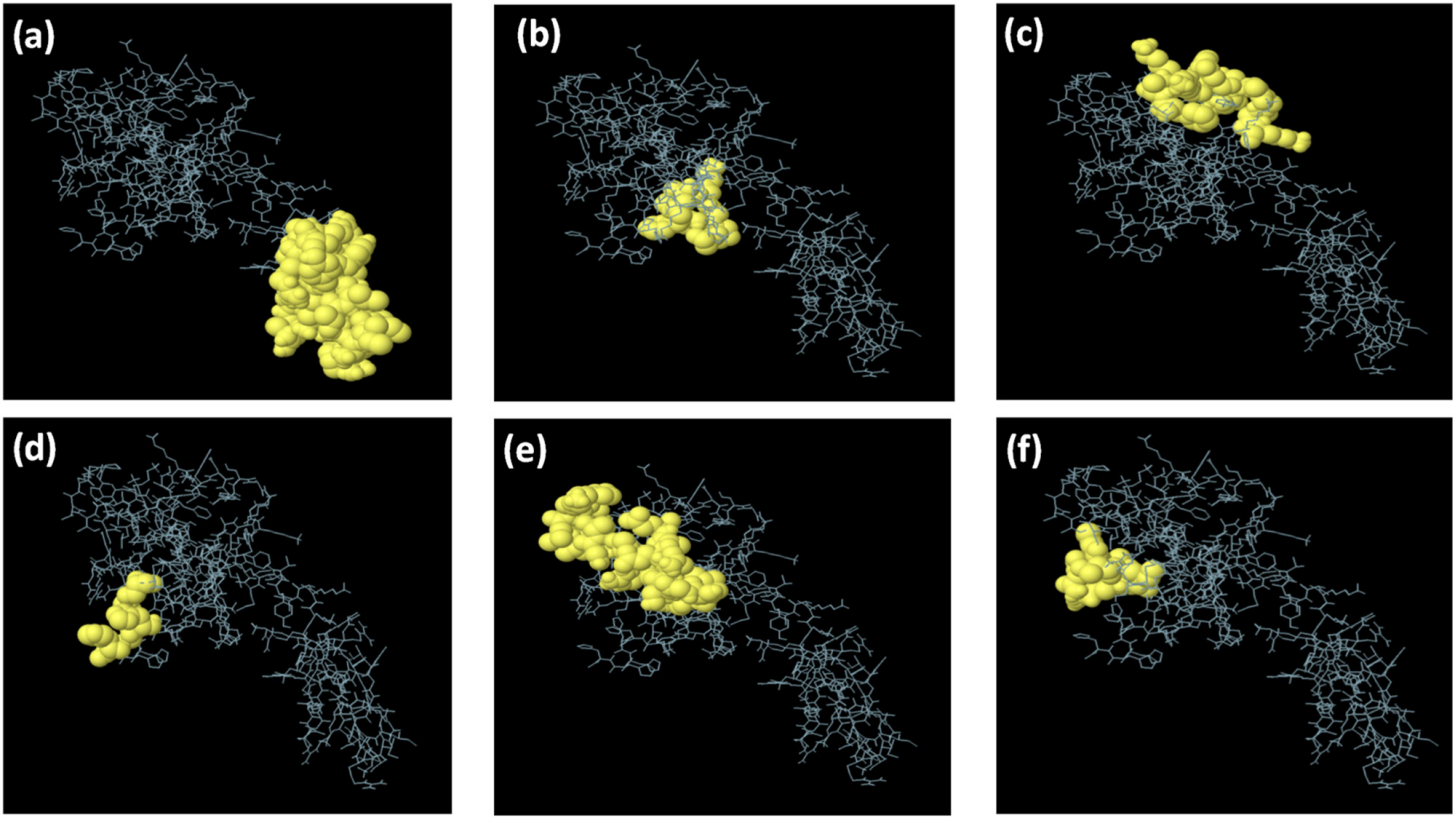
The yellow color highlighted residues indicate the discontinuous B-cell epitopes predicted in the designed vaccine constructs.

The ElliPro server predicted six discontinuous B-cell epitopes within the designed vaccine construct with varying lengths and scores, as shown in Figure 11. The highest-ranked epitope (score: 0.739) consists of 32 residues, indicating relatively higher accessibility for immune recognition. The second epitope (score: 0.717) and third epitope (score: 0.668) also showed notable potential for immune activation. Even the lower scoring of the fourth (4 residues, score: 0.639) and sixth (7 residues, score: 0.596) epitopes may contribute to antibody binding. These epitopes are expected to be capable of inducing a humoral immune response in the predicted vaccine, suggesting the potential effectiveness of the vaccine construct. Details of the predicted discontinuous B-cell epitopes, including residue composition, epitope length, and prediction scores, are summarized in Table 7.

### Disulphide engineering

**Figure 12:**
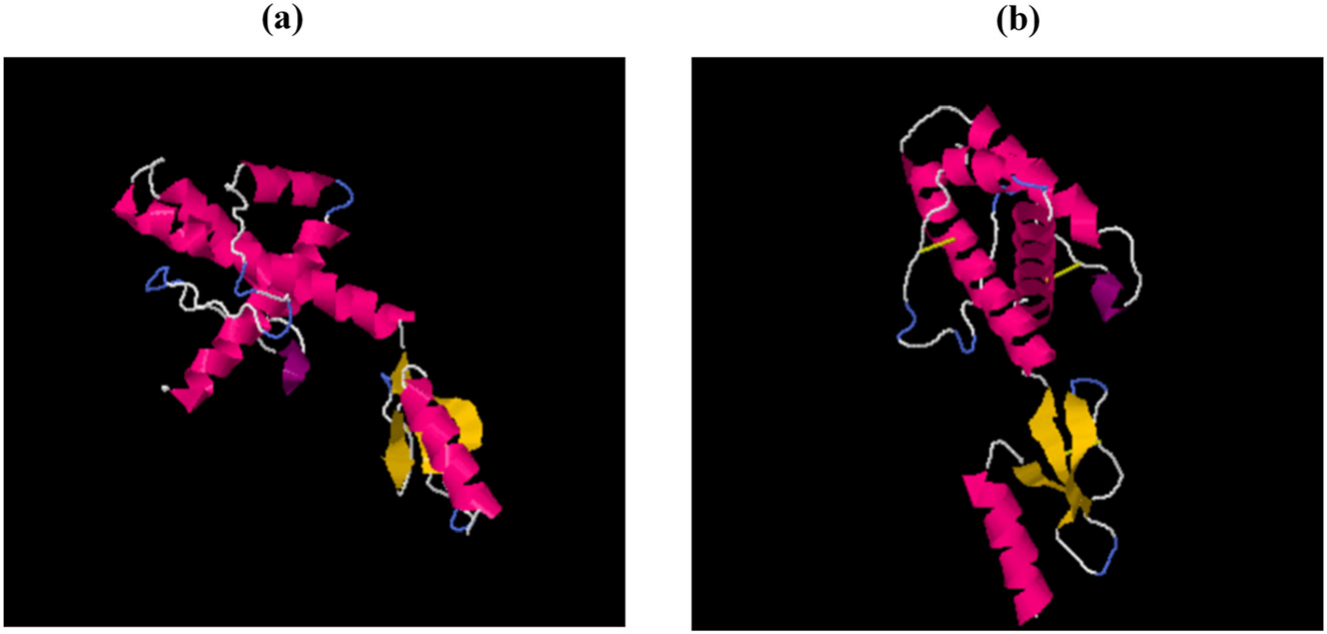
Disulfide engineering of the vaccine construct. (a) Original structure of the vaccine construct. (b) Engineered structure of the vaccine with two disulfide bonds shown in yellow.

Disulfide engineering was performed to enhance the structural stability of the vaccine construct by introducing cysteine mutations at selected residue pairs (Figure 12). Two disulfide bonds were designed at ALA60–ALA120 with a χ^3^ angle of +103.42 (1.04  kcal/mol) and VAL98–ALA150 with a χ^3^ angle of +106.14 (0.84  kcal/mol). The favorable χ^3^ angles imply proper dihedral geometry for disulfide bonding with minimal structural strain, and the low energy values indicate limited structural distortion. The introduction of these disulfide bonds may enhance structural stability and potentially improve immunogenicity of the vaccine, as protein rigidity is enhanced, potentially improving thermostability and resistance to degradation.

### Molecular docking and interaction analysis

Molecular docking was performed to evaluate the interaction between the designed multi-epitope vaccine construct and human innate immune receptors TLR2 and TLR8. Docking analysis showed favorable binding affinities, with the vaccine–TLR2 complex exhibiting a docking score of −1105.52 and the vaccine–TLR8 complex showing a score of −1152.9, suggesting the formation of stable complexes.

**Figure 13:**
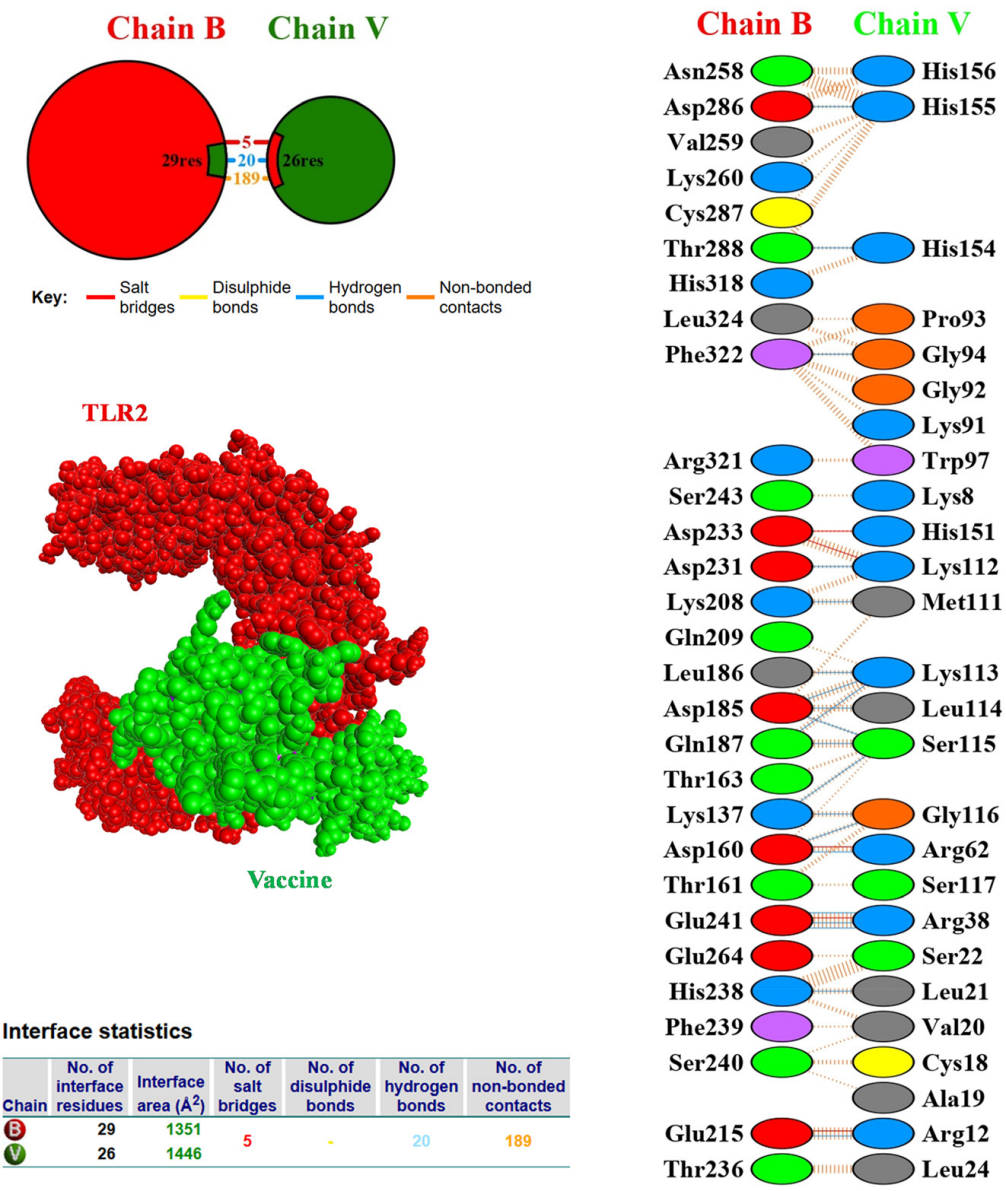
Intermolecular interaction network between the vaccine construct (chain V) and the TLR2 receptor (chain B), showing hydrogen bonds, salt bridges, and non-bonded contacts at the interface.

Detailed interaction analysis of the vaccine–TLR2 complex demonstrated extensive interface interactions mediated by multiple non-covalent interactions. The interface comprised 29 interacting residues from TLR2 (Chain B) and 26 interacting residues from the vaccine construct (Chain V), covering interface areas of 1351 Å^2^ and 1446 Å^2^, respectively (Figure 13). A total of 5 salt bridges, 20 hydrogen bonds, and 189 non-bonded contacts were identified, indicating substantial interface complementarity.

**Figure 14:**
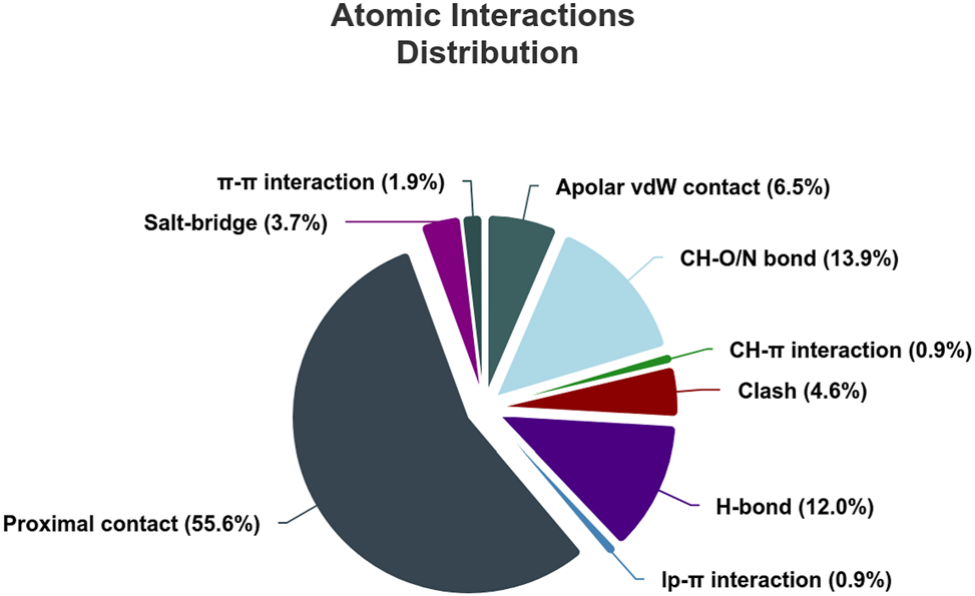
Distribution of atomic interactions in the vaccine–TLR2 complex as analyzed using CoCoMaps2.

Hydrogen bonding contributed to the stabilization of the vaccine–TLR2 interaction, involving residues such as ARG, HIS, LYS, GLY, SER, and LEU, with interaction distances ranging from 2.59 Å to 3.25 Å, as summarized in Table 8. In addition, electrostatic interactions, including salt bridges formed by residues such as ASP160–ARG62 and GLU215–ARG12, were observed at the interface. To further characterize the interface, CoCoMaps2 analysis was performed, which showed that proximal contacts (55.6 %), hydrogen bonds (12.0 %), CH–O/N bonds (13.9 %), and apolar van der Waals contacts (6.5 %) constituted the major interaction types (Figure 14). The buried surface area (BSA) analysis indicated a total buried area of 2803.3 Å^2^, with 58.73 % non-polar and 41.27 % polar contributions, reflecting a combination of hydrophobic and electrostatic interactions at the interface. Overall, these observations suggest that the vaccine construct forms a well-organized vaccine–TLR2 complex supported by multiple non-covalent interactions.

**Table 8:** Detailed hydrogen bonds in vaccine–TLR2 complex.

Sr. No.	TLR2	Vaccine	Type of bond	Distance (Å)
1	LYS137	SER115	Hydrogen bond	2.59
2	LYS137	GLY116	Hydrogen bond	2.73
3	ASP160	GLY116	Hydrogen bond	2.84
4	ASP160	ARG62	Hydrogen bond	2.81
5	ASP185	LYS113	Hydrogen bond	2.97
6	ASP185	LEU114	Hydrogen bond	2.93
7	ASP185	SER115	Hydrogen bond	3.25
8	LEU186	LYS113	Hydrogen bond	2.53
9	GLN187	LYS113	Hydrogen bond	2.57
10	GLN187	SER115	Hydrogen bond	2.86
11	LYS208	MET111	Hydrogen bond	2.64
12	GLU215	ARG12	Hydrogen bond	2.71
13	ASP231	LYS112	Hydrogen bond	2.76
14	HIS238	LEU21	Hydrogen bond	3.00
15	GLU241	ARG38	Hydrogen bond	2.63
16	GLU241	ARG38	Hydrogen bond	2.71
17	GLU241	ARG38	Hydrogen bond	2.71
18	ASP286	HIS155	Hydrogen bond	2.74
19	THR288	HIS154	Hydrogen bond	2.90
20	PHE322	GLY94	Hydrogen bond	2.77

**Figure 15:**
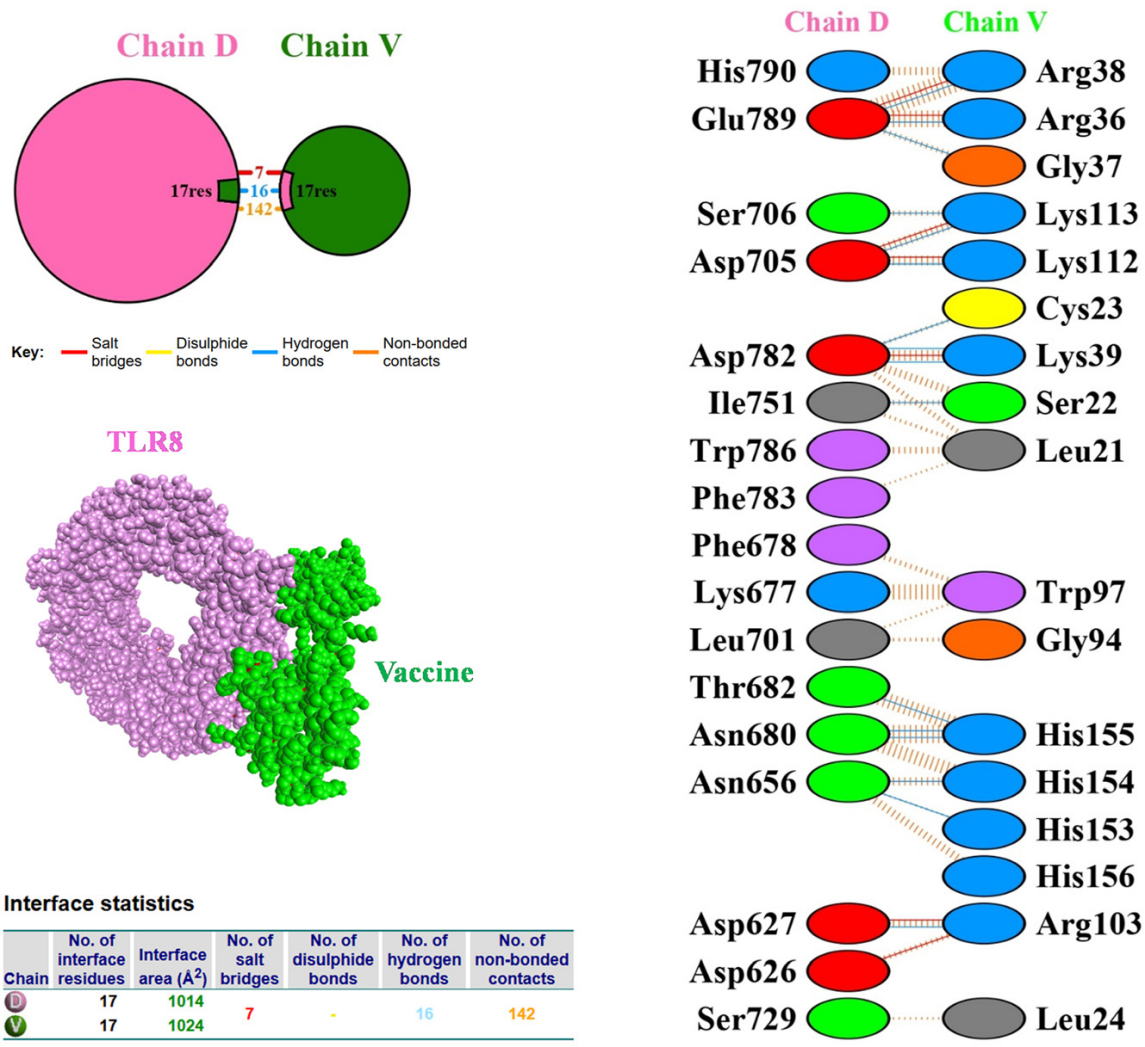
Intermolecular interactions between the vaccine construct (chain V) and the TLR8 receptor (chain D), highlighting hydrogen bonds, salt bridges, and non-bonded contacts at the interface.

In the case of the vaccine–TLR8 complex, interaction analysis showed a binding interface supported by multiple non-covalent interactions. A total of 16 hydrogen bonds with bond distances ranging from 2.47 Å to 3.25 Å, along with 7 salt bridges, were identified, indicating electrostatic contributions to the interaction. The interface comprised 17 residues from TLR8 (Chain D) and 17 residues from the vaccine construct (Chain V), covering surface areas of 1014 Å^2^ and 1024 Å^2^, respectively (Figure 15).

**Figure 16:**
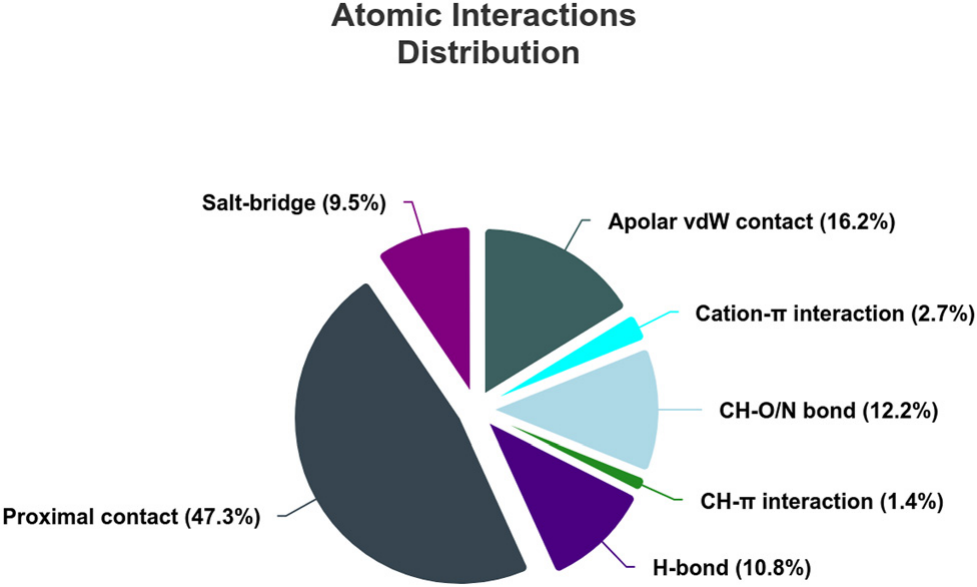
Distribution of atomic interactions in the vaccine–TLR8 complex as analyzed using CoCoMaps2.

Hydrogen bonding interactions involved residues such as ARG, HIS, LYS, ASN, and ASP, which are commonly involved in protein–protein interactions. Multiple hydrogen bond interactions observed between the same residue pairs correspond to distinct atomic interactions occurring at different bond distances. The interaction network was further supported by non-bonded and hydrophobic contacts, contributing to interface complementarity. The atomic interaction distribution analysis showed that proximal contacts (47.3 %), hydrogen bonds (10.8 %), CH–O/N bonds (12.2 %), and apolar van der Waals contacts (16.2 %) were the predominant interaction types (Figure 16).

Overall, these observations suggest that the vaccine construct forms a well-organized vaccine–TLR8 complex, which may facilitate interaction with TLR8-associated innate immune pathways. The detailed hydrogen bond interactions identified for the vaccine–TLR8 complex are summarized in Table 9.

**Table 9:** Detailed hydrogen bonds in vaccine-TLR8 complex.

Sr. No.	Vaccine	TLR8	Type of bond	Distance (Å)
1	ARG103	ASP627	Hydrogen bond	2.79
2	HIS153	ASN656	Hydrogen bond	2.94
3	HIS154	ASN656	Hydrogen bond	2.81
4	HIS155	ASN680	Hydrogen bond	2.89
5	HIS155	ASN680	Hydrogen bond	2.98
6	HIS155	THR682	Hydrogen bond	2.79
7	LYS112	ASP705	Hydrogen bond	2.56
8	LYS113	ASP705	Hydrogen bond	2.79
9	LYS113	SER706	Hydrogen bond	2.47
10	SER22	ILE751	Hydrogen bond	2.78
11	LYS39	ASP782	Hydrogen bond	2.67
12	CYS23	ASP782	Hydrogen bond	3.25
13	LYS39	ASP782	Hydrogen bond	2.54
14	ARG36	GLU789	Hydrogen bond	2.80
15	GLY37	GLU789	Hydrogen bond	2.92
16	ARG38	GLU789	Hydrogen bond	2.90

### Molecular dynamics simulations

**Figure 17:**
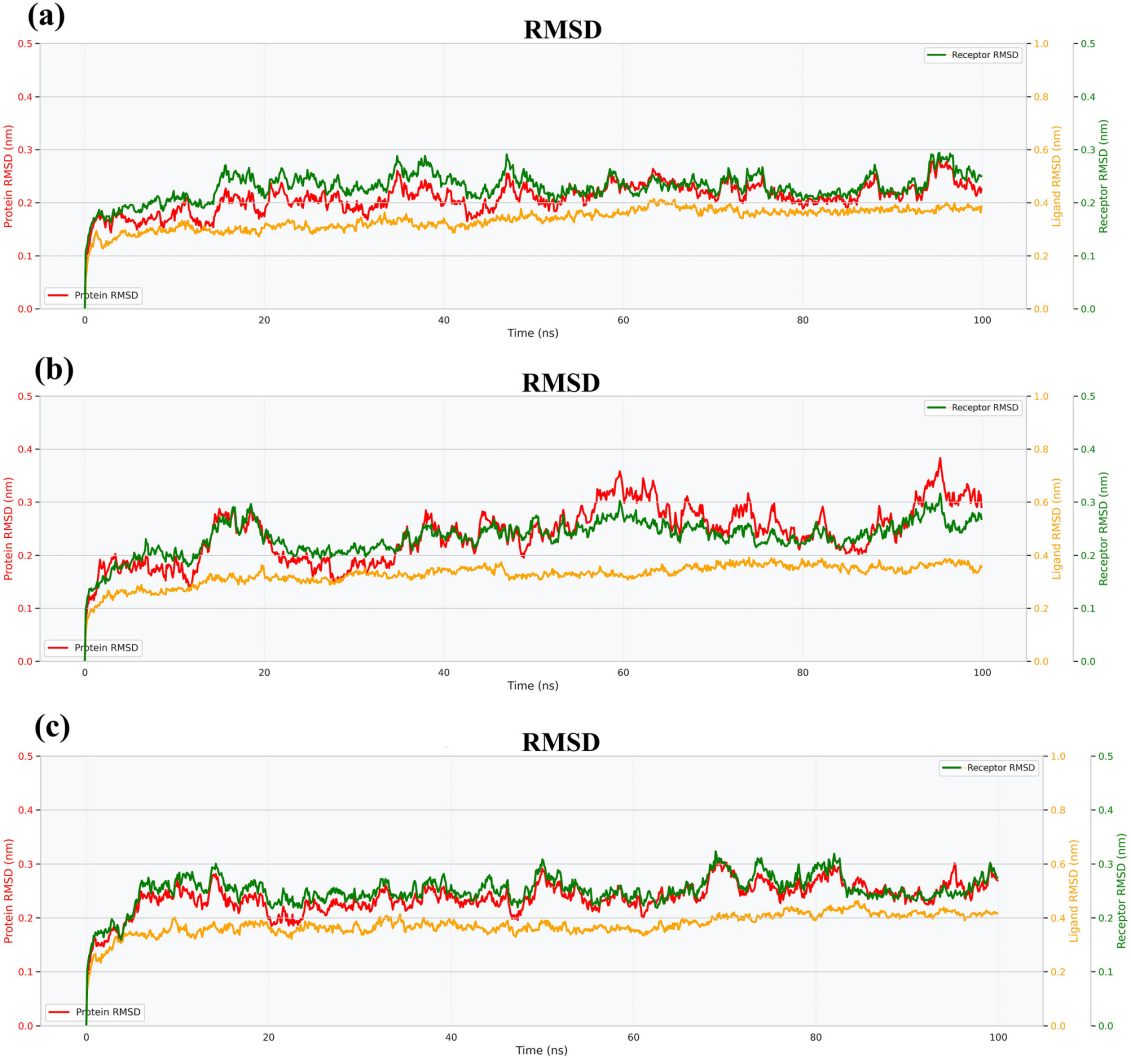
RMSD plots of the TLR2 receptor, vaccine construct, and TLR2–vaccine complex over 100 ns molecular dynamics simulations. The red curve represents the TLR2 receptor (labeled as “protein” in the graph), the yellow curve represents the vaccine construct (labeled as “ligand” in the graph), and the green curve represents the TLR2–vaccine complex (labeled as “receptor” in the graph). (a) Replica 1, (b) replica 2, and (c) replica 3.

The molecular dynamics simulations were performed for the vaccine–TLR2 complex. To improve the reliability of the analysis, three independent molecular dynamics simulation replicas were conducted for 100 ns each. The RMSD plots provide insight into the structural behavior and stability of the system across different replicas. In Replica 1 (Figure 17a), an initial increase in RMSD was observed within the first 10 ns, reaching approximately 0.15–0.18 nm, indicating early structural adaptation. From 10 to 50 ns, RMSD values stabilized within the range of 0.20–0.27 nm for the TLR2 receptor and 0.22–0.30 nm for the TLR2–vaccine complex, while the vaccine construct RMSD remained lower (0.14–0.20 nm), suggesting stable binding. Beyond 50 ns, the system maintained equilibrium with minor fluctuations, indicating structural stability of the complex.

In Replica 2 (Figure 17b), a similar equilibration phase was observed within the first 10 ns, with RMSD values reaching approximately 0.18–0.22 nm. During the mid-simulation phase, the TLR2 receptor RMSD increased to approximately 0.30–0.35 nm, with slightly higher fluctuations observed later, reaching up to ∼0.38 nm. However, the TLR2–vaccine complex RMSD remained relatively stable (0.25–0.30 nm), and the vaccine construct RMSD showed minimal deviation (0.15–0.20 nm), indicating maintained binding stability. In Replica 3 (Figure 17c), the system exhibited consistent behavior, with RMSD values increasing to approximately 0.15–0.20 nm during the initial equilibration phase. Throughout the simulation, TLR2 receptor RMSD remained within 0.22–0.30 nm, TLR2–vaccine complex RMSD within 0.25–0.32 nm, and vaccine construct RMSD within 0.16–0.22 nm, indicating a stable complex. Overall, all three simulation replicas demonstrated rapid equilibration within the first 10 ns, followed by stable RMSD profiles without significant structural drift. The RMSD values across the three replicas generally remained below 0.38 nm (3.8 Å), indicating overall structural stability. The observed fluctuations are attributed to the intrinsic flexibility of the multi-epitope vaccine construct, while the core interaction interface remained stable throughout the simulation.

**Figure 18:**
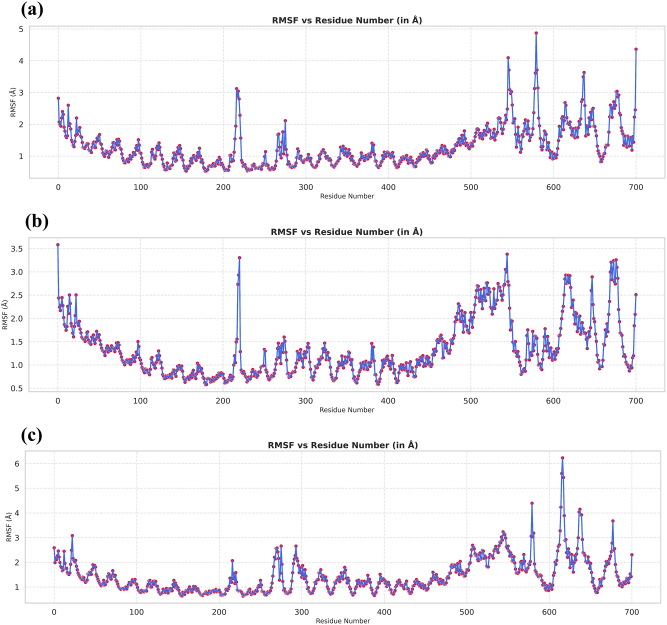
RMSF profiles of the vaccine–TLR2 complex over 100 ns molecular dynamics simulations from three independent replicas: (a) Replica 1, (b) replica 2, and (c) replica 3.

The RMSF analysis was performed to evaluate residue-level flexibility of the vaccine–TLR2 complex across three independent molecular dynamics simulation replicas. Residue numbering in the RMSF plots corresponds to the full complex, where residues 1–545 represent the TLR2 receptor and residues 546–701 correspond to the vaccine construct. This continuous residue numbering approach was used to maintain the structural context of the entire complex during analysis. In Replica 1 (Figure 18a), most residues exhibited relatively low fluctuations, with RMSF values predominantly within 0.8–2.0 Å, indicating stable structural regions. However, distinct peaks were observed around residue positions 200, 550–600, and 680–700, where RMSF values increased up to approximately 4–5 Å, suggesting localized flexibility.

In Replica 2 (Figure 18b), a similar fluctuation pattern was observed, with most residues showing RMSF values between 0.7 and 1.8 Å. Higher fluctuations were detected near residues 200 and 500–650, reaching approximately 3–3.5 Å, indicating moderate flexibility in these regions. Compared to Replica 1, the fluctuations were slightly lower, suggesting consistent dynamic behavior. In Replica 3 (Figure 18c), the RMSF profile showed comparable trends, with most residues fluctuating within 0.8–2.2 Å. Notable peaks were observed around residues 580–620, where RMSF values reached approximately 5–6 Å, representing the highest flexibility among the replicas. Additional moderate fluctuations were observed near residues 250–300 and 650–700. Across all three replicas, the majority of residues exhibited low to moderate RMSF values (0.8–2.0 Å), indicating that the core regions of the TLR2 receptor (residues 1–545) remain structurally stable. The highest and most frequent fluctuations observed beyond residue 545 correspond to the vaccine construct, particularly linker-associated segments, which are inherently flexible and facilitate conformational adaptability of the multi-epitope construct. Overall, the RMSF results demonstrate a consistent pattern of localized flexibility and global structural stability across all simulation replicas. These findings support the RMSD results, confirming that the vaccine–TLR2 complex maintains a stable interaction interface while allowing necessary conformational mobility.

**Figure 19:**
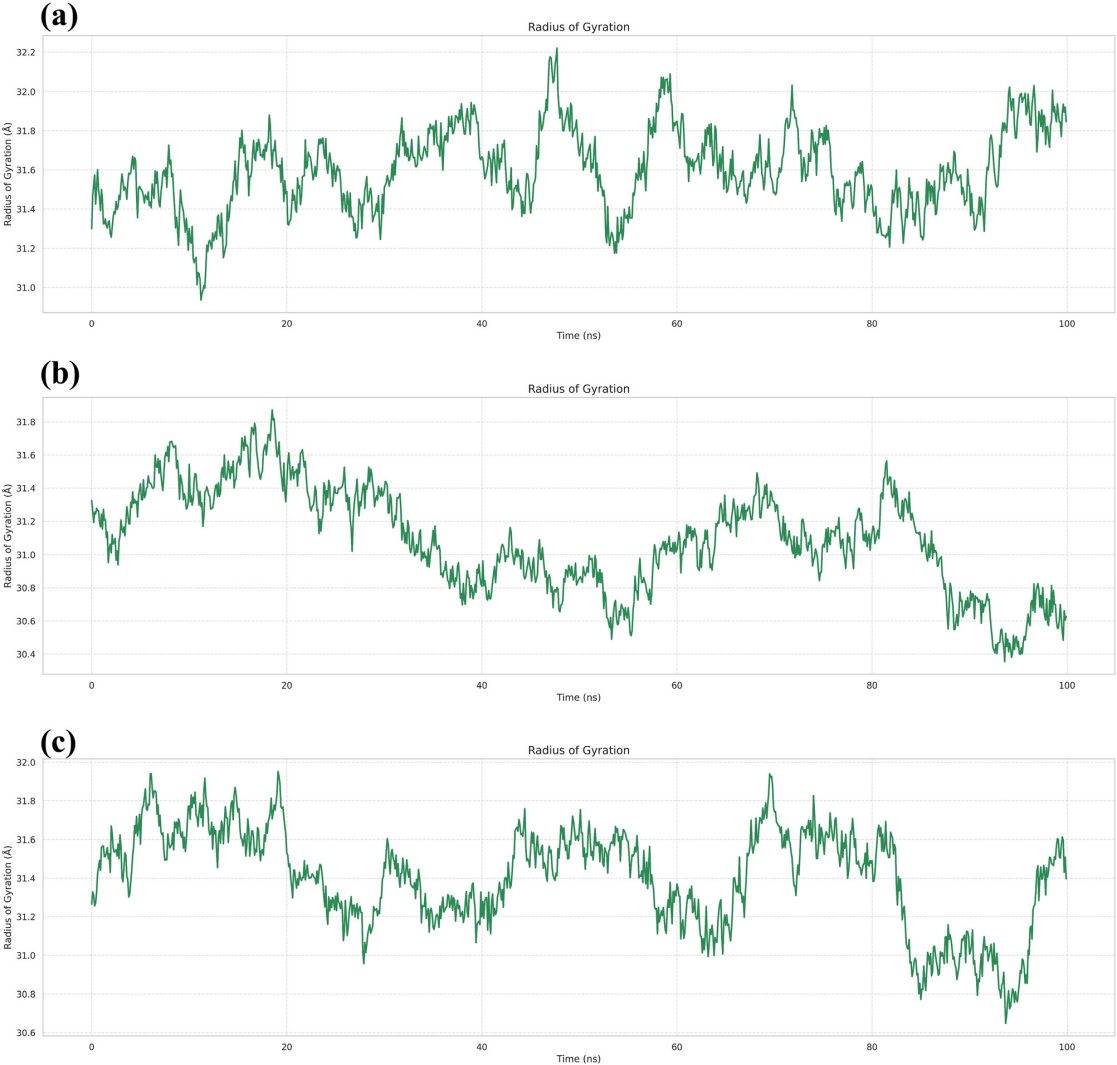
Radius of gyration (Rg) profiles of the vaccine–TLR2 complex over 100 ns molecular dynamics simulations from three independent replicas: (a) Replica 1, (b) replica 2, and (c) replica 3.

The radius of gyration (Rg) analysis was performed to evaluate the overall compactness and structural stability of the vaccine–TLR2 complex across three independent molecular dynamics simulation replicas. In Replica 1 (Figure 19a), the Rg values fluctuated within the range of approximately 31.0–32.2 Å throughout the simulation. Minor variations were observed, with occasional peaks near 32.2 Å, but no significant expansion or collapse of the structure was detected, indicating stable compactness. In Replica 2 (Figure 19b), the Rg values ranged between approximately 30.4–31.8 Å. A slight decrease in Rg was observed toward the later stages of the simulation, suggesting mild structural compaction; however, the overall profile remained stable without abrupt deviations. In Replica 3 (Figure 19c), the Rg values were maintained within approximately 30.7–32.0 Å, with moderate fluctuations throughout the simulation. A transient decrease around 80–90 ns was observed, followed by recovery, indicating temporary conformational adjustments rather than structural instability.

Across all three replicas, the Rg values remained within a narrow range of approximately 30.4–32.2 Å, demonstrating consistent compactness of the vaccine–TLR2 complex. The absence of sharp increases or continuous drift in Rg indicates that the complex did not undergo major unfolding or structural expansion during the simulations. Overall, the consistent Rg profiles across all replicas confirm that the vaccine–TLR2 complex maintains structural integrity and compactness throughout the simulation. The minor fluctuations observed are likely due to localized flexibility in linker and terminal regions, rather than global structural instability.

**Figure 20:**
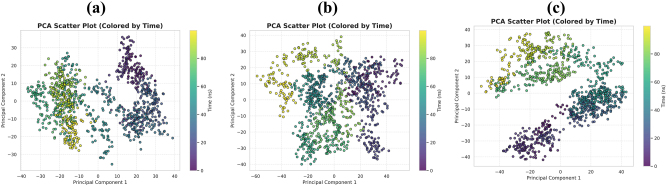
PCA plots of the vaccine–TLR2 complex from three independent molecular dynamics simulation replicas: (a) Replica 1, (b) replica 2, and (c) replica 3.

PCA was performed to evaluate the conformational dynamics of the vaccine–TLR2 complex across three independent molecular dynamics simulation replicas by projecting Cα atom motions onto the first two principal components, PC1 and PC2. In Replica 1 (Figure 20a), the conformational space showed relatively compact clustering with moderate dispersion along PC1, indicating stable structural behavior with limited large-scale motions. In Replica 2 (Figure 20b), a broader distribution of points was observed, particularly along PC1, suggesting increased conformational sampling and flexibility, consistent with the higher RMSD observed in this replica. In Replica 3 (Figure 20c), the distribution exhibited well-defined clusters with moderate dispersion, indicating stable and consistent dynamic behavior similar to Replica 1. Across all three replicas, distinct conformational clusters indicate that the system adopts multiple well-defined conformational states during the simulation. PC1 likely reflects the dominant collective motions, whereas PC2 captures secondary conformational fluctuations. The observed dispersion is mainly associated with flexible linker and terminal regions, whereas clustered regions indicate stability of the core structural framework. Overall, the PCA results demonstrate that the vaccine–TLR2 complex maintains structural coherence while exhibiting controlled conformational flexibility, consistent with RMSD, RMSF, and Rg analyses.

**Figure 21:**
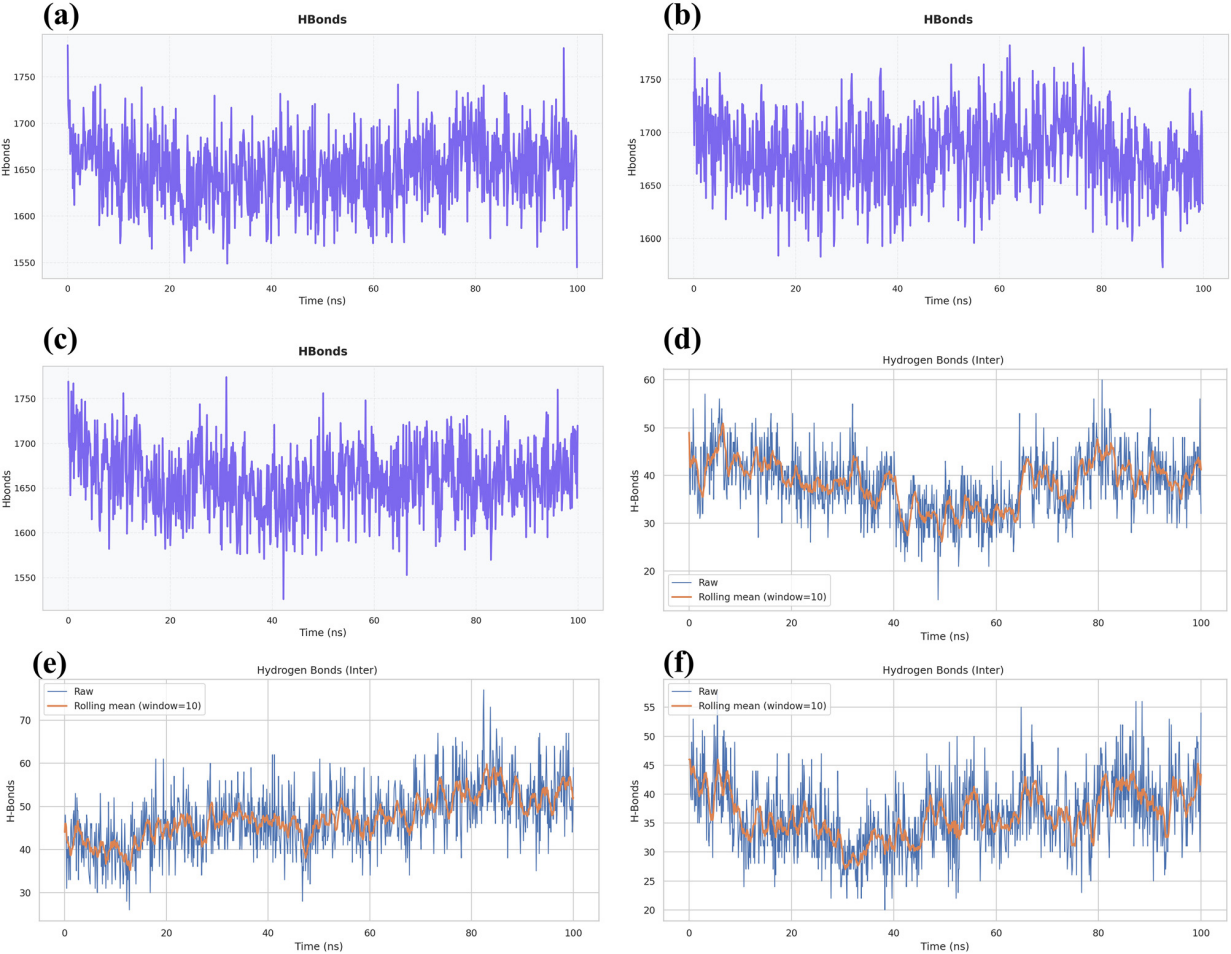
Hydrogen bond analysis of the vaccine–TLR2 complex during 100 ns molecular dynamics simulations. Overall hydrogen bonds of the vaccine–TLR2 complex during the simulation are shown for (a) replica 1, (b) replica 2, and (c) replica 3. Intermolecular hydrogen bonds between the vaccine–TLR2 complex are shown for (d) replica 1, (e) replica 2, and (f) replica 3.

Hydrogen bond analysis was performed to evaluate the stability and interaction strength of the vaccine–TLR2 complex across three independent molecular dynamics simulation replicas (Figure 21a–f). Both total hydrogen bonds and intermolecular hydrogen bonds between the vaccine construct and TLR2 receptor were analyzed. In Replica 1 (Figure 21a), the total number of hydrogen bonds fluctuated between approximately 1600–1750, indicating stable intramolecular interactions within the complex. The intermolecular hydrogen bonds (Figure 21d) ranged between 30 and 50, with an average of approximately 40–45 bonds, showing consistent interaction between the vaccine and receptor throughout the simulation. In Replica 2 (Figure 21b), total hydrogen bonds remained within approximately 1600–1780, demonstrating stable structural integrity. The intermolecular hydrogen bonds (Figure 21e) showed slightly higher values compared to Replica 1, fluctuating between 35 and 60 bonds, with an average around 45–50 bonds, suggesting strong and persistent binding interactions. In Replica 3 (Figure 21c), total hydrogen bonds varied within approximately 1550–1750, showing stable behavior similar to the other replicas.

**Figure 22:**
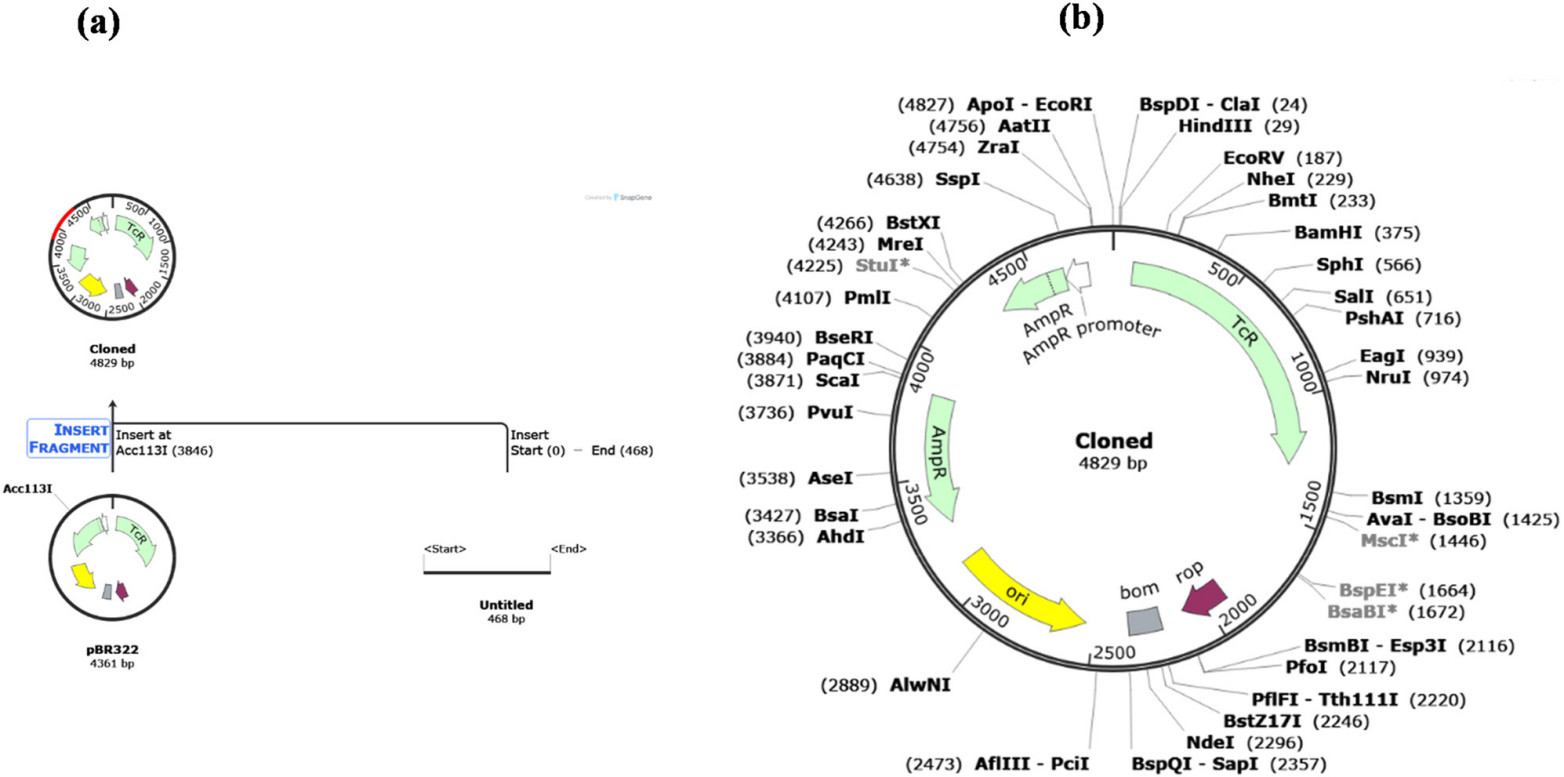
*In silico* cloning of the optimized vaccine sequence into the pBR322 expression vector. (a) Recombinant plasmid showing insertion at the AccI restriction site. (b) Final construct indicating vector size after insertion.

The intermolecular hydrogen bonds (Figure 21f) ranged between 25 and 55 bonds, with an average of approximately 35–45 bonds, indicating stable but slightly more variable interactions. Across all three replicas, the total hydrogen bonds remained consistently high with no significant loss over time, confirming that the structural integrity of the vaccine–TLR2 complex is maintained during the simulation. The intermolecular hydrogen bond profiles further demonstrate stable and persistent interactions at the binding interface, with average values ranging between 35 and 50 hydrogen bonds. Overall, the consistent hydrogen bond patterns across all replicas indicate that the vaccine construct forms stable and sustained interactions with the TLR2 receptor. Minor fluctuations observed during the simulation are attributed to local conformational flexibility, particularly in linker regions, rather than disruption of the binding interface. These findings further support the RMSD, RMSF, and Rg results, confirming the dynamic stability of the vaccine–TLR2 complex.

### *In silico* cloning

Initially, the amino acid sequence of the vaccine construct was reverse translated into a nucleotide sequence with a length of 468 nucleotides, followed by codon optimization to improve its expression in *E. coli* strain K12. Furthermore, cloning was performed using the pBR322 vector with the help of SnapGene software. The vaccine sequence was inserted at the AccI restriction site of the pBR322 vector, as shown in Figure 22a. Initially, the size of the vector was 4361 bp, and after insertion of the vaccine fragment, the total size of the vector became 4829 bp, as shown in Figure 22b.

### Immune simulation

**Figure 23:**
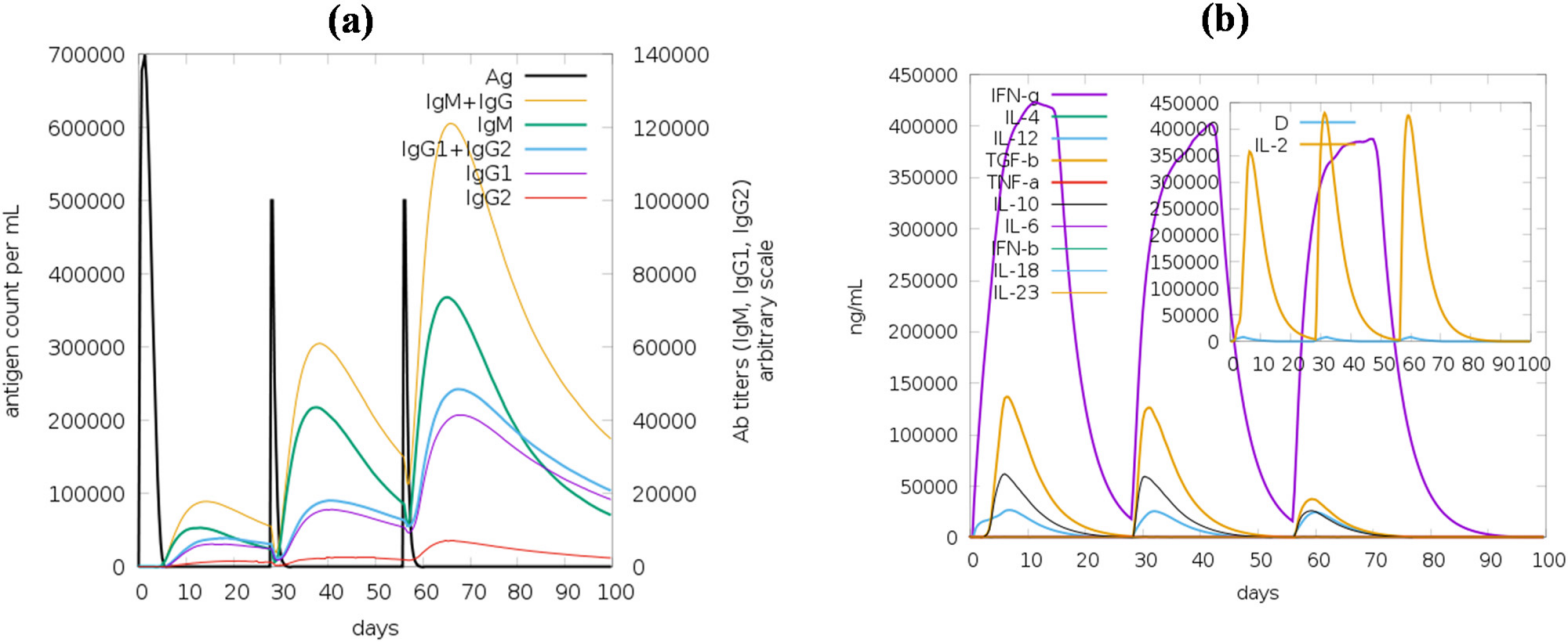
Immune simulation results. (a) Antigen and antibody responses over time. (b) Cytokine profiles illustrating immune response dynamics.

The immune simulation of the multi-epitope vaccine construct was performed using a three-dose regimen administered on days 1, 28, and 56. Each dose showed an increase in antigen levels, as shown in Figure 23a, followed by humoral and cellular immune responses. The vaccine construct induced IgM responses during the primary phase, while IgG1 responses increased after the second and third doses, suggesting the development of immunological memory. Cell-mediated immune responses were indicated by increased concentrations of IFN-γ and IL-2, as shown in Figure 23b, suggesting a Th1-biased immune response. Intermediate levels of IL-12, IL-18, and IFN-β were observed, which may contribute to antiviral responses, along with regulatory cytokines such as IL-10 and TGF-β that are associated with immune homeostasis. The observed antibody and cytokine profiles suggest that the vaccine construct may induce antigen-specific immune responses with recall potential upon booster doses.

**Figure 24:**
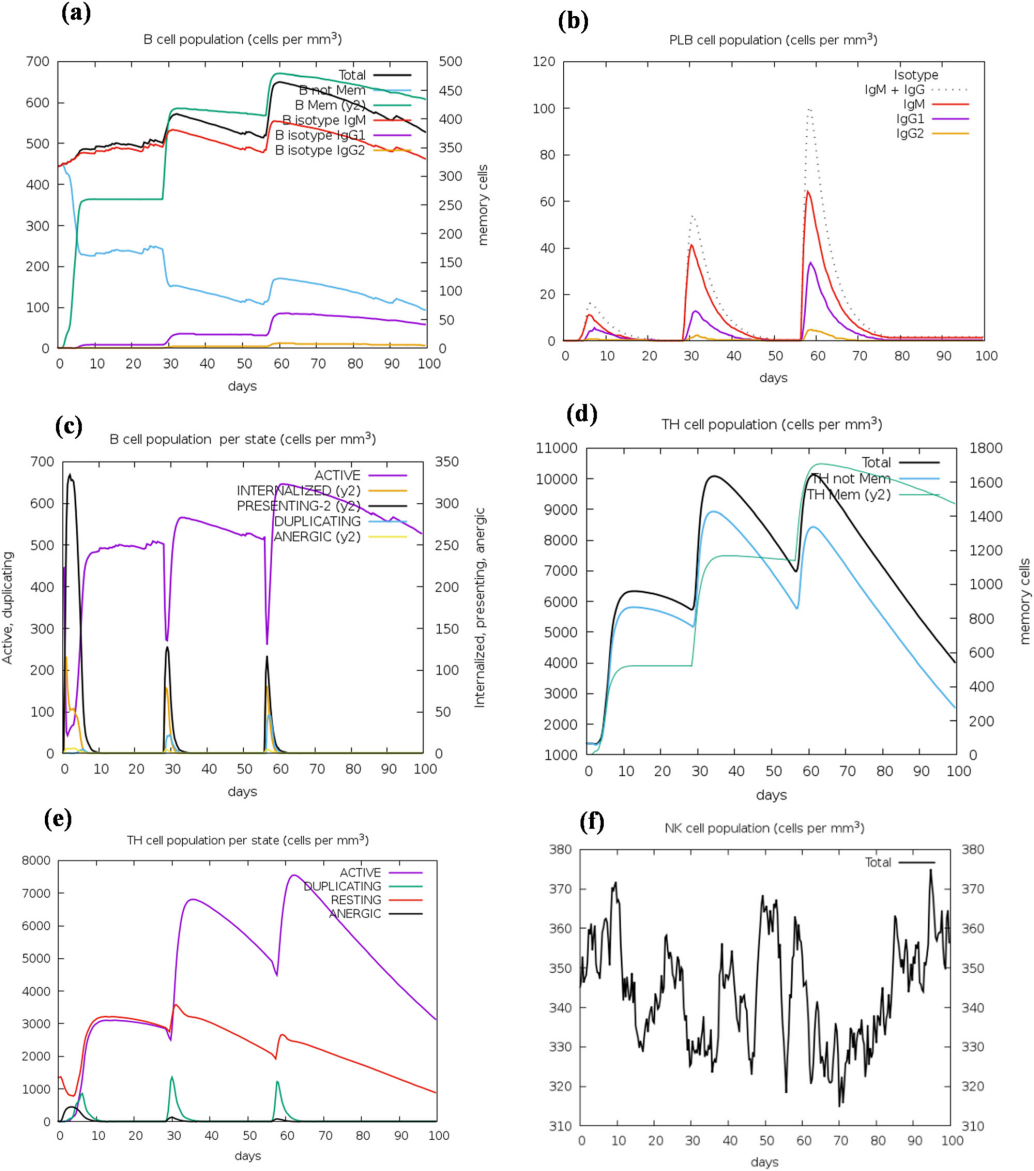
Immune cell population dynamics during simulation. (a) Total B-cell population. (b) Plasma B-cell population. (c) B-cell population by state. (d) Cytotoxic T-cell population. (e) Cytotoxic T-cell population by state. (f) Natural killer cell population.

A rise in total B-cell population and the formation of memory B-cells were observed after the first dose (day 1), as shown in Figure 24a. IgM was the dominant isotype during this phase, with plasma B-cells secreting IgM peaking around day 10 (Figure 24b). There was also an increase in T helper (Th) cells, along with early memory Th cell formation. A peak in IFN-γ levels was observed, suggesting a Th1-associated response. After the second dose (day 28), an enhanced response was observed in both B-cell and T-cell populations (Figure 24c). Memory B-cells increased further, and IgG1-producing plasma B-cells showed higher levels compared to IgM. The Th cells expanded, along with an increase in memory Th cells. This dose resulted in a stronger recall response and was associated with increased cytokine production, particularly IL-2 and IFN-γ, suggesting enhanced cellular immune responses. After the third dose (day 56), higher levels of immune responses were observed. Antibody-secreting B-cells and Th cells reached their peak levels (Figure 24d), along with increased memory Th cell populations (Figure 24e). Increased IgG1 and IgG2 responses were observed among plasma cells, indicating class switching. Repeated increases in effector and memory Th and B-cell populations suggest the development of immunological memory. The number of NK cells remained relatively consistent throughout all doses (Figure 24f), indicating balanced immune activation.

## Discussion

In the present study, we found that the *in silico* designed multi-epitope vaccine targeting the yellow fever virus envelope glycoprotein exhibited predicted antigenicity, non-allergenicity, and the ability to elicit both B-cell and T-cell immune responses. The vaccine construct was developed using immunoinformatics approaches, focusing on the envelope glycoprotein, which plays a key role in viral binding and entry. The selected epitopes were identified as non-allergenic, non-toxic, and capable of inducing both humoral and cellular immune responses. These findings suggest a potential improvement compared to classical vaccine approaches for YFV, particularly the 17D live-attenuated vaccine, which is effective but requires caution in immunocompromised individuals, pregnant women, and infants under six months [64]. Unlike the 17D vaccine, which has been associated with rare but severe adverse effects such as Yellow Fever Vaccine-Associated Viscerotropic Disease and Yellow Fever Vaccine-Associated Neurotropic Disease [64], the *in silico* vaccine designed in this study does not contain live viral components, Consequently, it may reduce risks associated with live-attenuated vaccines; however, experimental studies are required to validate its safety and efficacy.

While traditional approaches have long been used for vaccine development, they are often costly and time-consuming. Reverse vaccinology offers an alternative strategy by identifying and prioritizing antigenic proteins through genome and proteome analysis, which may facilitate vaccine formulation [65]. Compared to previous studies, such as the study by Tosta et al. [65], the present study additionally incorporates phylogenetic and conservation analysis, supporting the evolutionary stability of the YFV envelope glycoprotein. The vaccine construct design incorporated specific linkers, including KK linkers for B-cell epitopes, AAY linkers for MHC-I epitopes, and GPGPG linkers for MHC-II epitopes. The primary objective of docking the vaccine construct with Toll-like receptors was to explore potential interaction patterns and immunostimulatory capacity rather than to establish definitive receptor specificity. Such immunoinformatics analyses provide preliminary insights into how the vaccine may interact with innate immune pathways involved in shaping adaptive immune responses [66]. TLR2 and TLR8 are well-established pattern recognition receptors (PRRs) involved in antiviral immunity [67]. TLR2, a cell-surface pattern-recognition receptor, participates in innate immune responses to viral components and activates downstream MyD88-dependent NF-κB and MAPK signaling pathways, leading to the production of pro-inflammatory cytokines [68], 69]. Although the selected adjuvant does not represent a classical ligand with a well-defined TLR2 recognition motif, several studies have shown that peptide-based and defensin-like molecules can indirectly modulate TLR2-mediated signaling. Therefore, docking with TLR2 was conducted as an exploratory assessment to evaluate potential innate immune engagement rather than direct receptor specificity [67].

In addition, TLR8, an endosomal receptor, plays a critical role in antiviral defense by recognizing viral single-stranded RNA and inducing type I interferon (IFN-α/β) responses. The inclusion of TLR8 in the docking analysis was intended to assess the vaccine construct’s potential to interact with receptors relevant to antiviral immune signaling pathways. The docking results revealed favorable ClusPro interaction scores for both TLR2 and TLR8, supporting the possibility of stable receptor engagement. Similar interaction trends involving these receptors have been reported in previous immunoinformatics studies on antiviral vaccine constructs [70], 71]. However, these findings should be considered preliminary, and experimental validation is required to confirm receptor-specific activation and downstream immune responses.

This study also includes the prediction of discontinuous B-cell epitopes, whereas the YFV vaccine design study by Silva et al. [72] focused on linear B-cell epitope prediction. Discontinuous B-cell epitopes contribute to humoral immune responses and long-term immunity, as they consider the three-dimensional structure of the protein, enabling interaction with B-cell receptors in a manner similar to natural infection [73], 74].

In this study, the vaccine–TLR2 complex exhibited RMSD values in the range of approximately 2–3.8 Å across three independent molecular dynamics simulation replicas and reached equilibrium after approximately 10–20 ns, indicating stable binding with moderate flexibility. Similar behavior has been reported in a previous study by Sharma et al., where the vaccine–receptor complex showed higher RMSD values with an average of 12.02 ± 1.52 Å, while the vaccine construct alone reached 16.87 ± 2.20 Å [75]. These deviations were attributed to flexible loops and linker regions within multi-epitope constructs, while the systems remained stable after equilibration, indicating that elevated RMSD values reflect structural flexibility rather than instability. Notably, Sharma et al. performed molecular dynamics simulations using multiple replicas; accordingly, the present study adopted a similar approach by conducting three independent simulation replicas to enhance the reliability and reproducibility of the findings. Furthermore, Sharma et al. evaluated hydrogen bond interactions and buried surface area to assess interface stability, which is consistent with the analyses performed in the present study [75]. In contrast, the present study utilized the AMBER simulation suite, and due to computational constraints, time-resolved buried surface area analysis during molecular dynamics simulations was not performed, which may limit the robustness and reproducibility of the findings. However, the buried surface area of the vaccine–TLR2 complex was calculated using the CoCoMaps2 web server, supporting the stability of the interaction. Immune response simulations indicated that the vaccine construct may elicit adaptive immune responses, including increases in IgM, IgG1, and IgG2 antibodies, along with activation of CD4+ helper T-cells and CD8+ cytotoxic T-cells. These findings suggest the potential of the vaccine construct to induce immune responses against yellow fever virus.

Despite these findings, several limitations should be considered. First, this study is entirely computational and lacks experimental validation through *in vitro* or *in vivo* approaches. Computational predictions, including antigenicity, epitope binding, immune simulations, and molecular docking, provide preliminary insights but do not fully represent biological complexity. Although buried surface area analysis was performed using CoCoMaps2, additional interface characterization and experimental validation would further strengthen the finding. Moreover, the stability and expression of the vaccine construct were evaluated computationally, and experimental validation is required. Population coverage analysis also indicated lower HLA representation in certain regions, suggesting that further epitope refinement may be necessary. Furthermore, post-translational modifications, immunodominance variations, and potential adverse effects remain unexplored. Therefore, experimental validation, including *in vitro* assays, animal studies, and clinical investigations, will be necessary to evaluate the safety and immunogenicity of the proposed vaccine construct.

## Conclusions

This study presents the computational design of a multi-epitope vaccine against yellow fever virus. The construct demonstrated predicted immunogenicity, safety, and global population coverage, along with favorable interactions with TLR2 and TLR8. Immune simulations suggested its potential to induce adaptive immune responses involving memory cells and cytokine production. The vaccine design addresses some limitations associated with live-attenuated vaccines by targeting conserved viral regions. However, these findings are based on computational analyses, and experimental validation is required to confirm efficacy and safety. Future studies should focus on *in vitro* expression, immunogenicity evaluation, and *in vivo* validation to further assess the potential of the designed vaccine.

## References

[1] Srivastava S, Jayaswal N, Gupta P, Sridhar SB, Jaiswal P, Tariq M (2026). The yellow fever vaccine journey: milestones and future directions. Vaccines.

[2] Hewson R (2024). Understanding viral haemorrhagic fevers: virus diversity, vector ecology, and public health strategies. Pathogens.

[3] Modrow S, Falke D, Truyen U, Schätzl H (2013). Viruses with single-stranded, positive-sense RNA genomes. Molecular virology.

[4] Srivastava S, Dhoundiyal S, Kumar S, Kaur A, Khatib MN, Gaidhane S (2024). Yellow fever: global impact, epidemiology, pathogenesis, and integrated prevention approaches. Infezioni Med Le.

[5] Rana A, Akhter Y (2016). A multi-subunit based, thermodynamically stable model vaccine using combined immunoinformatics and protein structure based approach. Immunobiology.

[6] Tuells J, Henao-Martínez AF, Franco-Paredes C (2022). Yellow fever: a perennial threat. Arch Med Res.

[7] Johansson MA, Arana-Vizcarrondo N, Biggerstaff BJ, Staples JE (2010). Incubation periods of yellow fever virus. Am J Trop Med Hyg.

[8] Monath TP, Barrett ADT (2003). Pathogenesis and pathophysiology of yellow fever. Adv Virus Res.

[9] Monath TP, Vasconcelos PFC (2015). Yellow fever. J Clin Virol.

[10] Ho Y-L, Joelsons D, Leite GFC, Malbouisson LMS, Song ATW, Perondi B (2019). Severe yellow fever in Brazil: clinical characteristics and management. J Trav Med.

[11] Kotar SL, Gessler JE (2017). Yellow fever: a worldwide history: McFarland.

[12] Duarte‐Neto AN, Cunha MP, Marcilio I, Song ATW, de Martino RB, Ho YL (2019). Yellow fever and orthotopic liver transplantation: new insights from the autopsy room for an old but re‐emerging disease. Histopathology.

[13] Giugni FR, Aiello VD, Faria CS, Pour SZ, dos Passos Cunha M, Giugni MV (2023). Understanding yellow fever-associated myocardial injury: an autopsy study. EBioMedicine.

[14] Reno E, Quan NG, Franco-Paredes C, Chastain DB, Chauhan L, Rodriguez-Morales AJ (2020). Prevention of yellow fever in travellers: an update. Lancet Infect Dis.

[15] Nwaiwu AU, Musekiwa A, Tamuzi JL, Sambala EZ, Nyasulu PS (2021). The incidence and mortality of yellow fever in Africa: a systematic review and meta-analysis. BMC Infect Dis.

[16] Wakil I (2014). Spread of hemorrhagic/infectious diseases. African Tradit Herb Res Clin Newslett.

[17] Possas C, Lourenço-de-Oliveira R, Tauil PL, Pinheiro FP, Pissinatti A, Cunha RV (2018). Yellow fever outbreak in Brazil: the puzzle of rapid viral spread and challenges for immunisation. Mem Inst Oswaldo Cruz.

[18] Hansen CA, Barrett ADT (2021). The present and future of yellow fever vaccines. Pharmaceuticals.

[19] Le Hir A, Durand GA, Boucraut J, Garnier A, Mura M, Diamantis S (2024). Yellow fever vaccine-associated neurologic and viscerotropic disease: a 10-year case series of the French National Reference Center for arboviruses with clinical and immunological insights. J Trav Med.

[20] Ferrara P, Masuet-Aumatell C, Ramon-Torrell JM (2021). Acceptance of yellow fever vaccine in the older traveller: a cohort study. Acta Biomed: Atenei Parmensis.

[21] Porudominsky R, Gotuzzo EH (2018). Yellow fever vaccine and risk of developing serious adverse events: a systematic review. Rev Panam Salud Públic.

[22] Soleymani S, Tavassoli A, Housaindokht MR (2022). An overview of progress from empirical to rational design in modern vaccine development, with an emphasis on computational tools and immunoinformatics approaches. Comput Biol Med.

[23] (2025). UniProt: the universal protein knowledgebase in 2025. Nucleic Acids Res.

[24] Doytchinova IA, Flower DR (2007). VaxiJen: a server for prediction of protective antigens, tumour antigens and subunit vaccines. BMC Bioinf.

[25] Dimitrov I, Bangov I, Flower DR, Doytchinova I (2014). AllerTOP v. 2—a server for in silico prediction of allergens. J Mol Model.

[26] Naveed M, Asim M, Aziz T, Saqi MM, Rehman HM, Al-Megrin WAI (2025). Development of a broad-spectrum multiepitope vaccine against dabie bandavirus through immunoinformatic approaches. Int Immunopharmacol.

[27] Larsen JE, Lund O, Nielsen M (2006). Improved method for predicting linear B-cell epitopes. Immunome Res.

[28] Lundegaard C, Lund O, Nielsen M (2008). Accurate approximation method for prediction of class I MHC affinities for peptides of length 8, 10 and 11 using prediction tools trained on 9mers. Bioinformatics.

[29] Jensen KK, Andreatta M, Marcatili P, Buus S, Greenbaum JA, Yan Z (2018). Improved methods for predicting peptide binding affinity to MHC class II molecules. Immunology.

[30] Naveed M, Toheed M, Aziz T, Asim M, Qadir P, Rehman HM (2025). Rational computational design and development of an immunogenic multiepitope vaccine incorporating transmembrane proteins of Fusobacterium necrophorum. Sci Rep.

[31] Yu C, Wu Q, Xin J, Yu Q, Ma Z, Xue M (2024). Designing a smallpox B-cell and T-cell multi-epitope subunit vaccine using a comprehensive immunoinformatics approach. Microbiol Spectr.

[32] Bui HH, Sidney J, Li W, Fusseder N, Sette A (2007). Development of an epitope conservancy analysis tool to facilitate the design of epitope-based diagnostics and vaccines. BMC Bioinf.

[33] Bui HH, Sidney J, Dinh K, Southwood S, Newman MJ, Sette A (2006). Predicting population coverage of T-cell epitope-based diagnostics and vaccines. BMC Bioinf.

[34] Shen Y, Maupetit J, Derreumaux P, Tufféry P (2014). Improved PEP-FOLD approach for peptide and miniprotein structure prediction. J Chem Theor Comput.

[35] Kozakov D, Hall DR, Xia B, Porter KA, Padhorny D, Yueh C (2017). The ClusPro web server for protein–protein docking. Nat Protoc.

[36] Moin AT, Patil RB, Tabassum T, Araf Y, Ullah MA, Snigdha HJ (2022). Immunoinformatics approach to design novel subunit vaccine against the Epstein-Barr virus. Microbiol Spectr.

[37] Dey J, Mahapatra SR, Singh PK, Prabhuswamimath SC, Misra N, Suar MJI (2023). Designing of multi-epitope peptide vaccine against Acinetobacter baumannii through combined immunoinformatics and protein interaction–based approaches. ..

[38] Tarrahimofrad H, Rahimnahal S, Zamani J, Jahangirian E, Aminzadeh SJSR (2021). Designing a multi-epitope vaccine to provoke the robust immune response against influenza A H7N9. ..

[39] Naveed M, Husnain M, Aziz T, Qadir P, Asim M, Majeed MN (2026). Immunoinformatics-based design and evaluation of a multi-epitope vaccine against Vibrio fluvialis. Sci Rep.

[40] Asim M, Makki HH (2026). Molecular characterization and immunoinformatics-based design of a multi-epitope vaccine against Staphylococcus nepalensis. Antonie Leeuwenhoek.

[41] Gasteiger E, Hoogland C, Gattiker A, Duvaud S, Wilkins MR, Appel RD (2005). Protein identification and analysis tools on the ExPASy server. The proteomics protocols handbook.

[42] Hanif N, Arshad S, Asim M, Nadeem AS, ur Rehman T, Shafique N (2024). In silico characterization of hypothetical protein AZJ53_10480 in Streptococcus pneumoniae. BioSci Rev.

[43] Hon J, Marusiak M, Martinek T, Kunka A, Zendulka J, Bednar D (2021). SoluProt: prediction of soluble protein expression in Escherichia coli. Bioinformatics.

[44] Buchan DWA, Moffat L, Lau A, Kandathil Shaun M, Jones DT (2024). Deep learning for the PSIPRED protein analysis workbench. Nucleic Acids Res.

[45] Araf Y, Moin AT, Timofeev VI, Faruqui NA, Saiara SA, Ahmed N (2022). Immunoinformatic design of a multivalent peptide vaccine against mucormycosis: targeting FTR1 protein of major causative fungi. Front Immunol.

[46] Abramson J, Adler J, Dunger J, Evans R, Green T, Pritzel A (2024). Accurate structure prediction of biomolecular interactions with AlphaFold 3. Nature.

[47] Ko J, Park H, Heo L, Seok C (2012). GalaxyWEB server for protein structure prediction and refinement. Nucleic Acids Res.

[48] Hanif N, Arif AAA, Ali SAS, Anees MAM, Anees MAM, Arshad A (2025). Computational drug design targeting MYH7 for hypertrophic cardiomyopathy integrating molecular docking, density functional theory, and molecular dynamics simulations. J Appl Biol Sci.

[49] Laskowski R, MacArthur M, Thornton J (2006). PROCHECK: validation of protein-structure coordinates.

[50] Colovos C, Yeates TO (1993). Verification of protein structures: patterns of nonbonded atomic interactions. Protein Sci : a publication of the Protein Society.

[51] Wiederstein M, Sippl MJ (2007). ProSA-web: interactive web service for the recognition of errors in three-dimensional structures of proteins. Nucleic Acids Res.

[52] Waterhouse AM, Studer G, Robin X, Bienert S, Tauriello G, Schwede T (2024). The structure assessment web server: for proteins, complexes and more. Nucleic Acids Res.

[53] Ponomarenko J, Bui HH, Li W, Fusseder N, Bourne PE, Sette A (2008). ElliPro: a new structure-based tool for the prediction of antibody epitopes. BMC Bioinf.

[54] Craig DB, Dombkowski AA (2013). Disulfide by design 2.0: a web-based tool for disulfide engineering in proteins. BMC Bioinf.

[55] Amin RN, Moin AT, Patil R, Barketullah Robin T, Zubair T, Nawal N (2023). Designing a polyvalent vaccine targeting multiple strains of varicella zoster virus using integrated bioinformatics approaches. Front Microbiol.

[56] Laskowski RA (2022). PDBsum 1: a standalone program for generating PDBsum analyses. Protein Sci.

[57] Chawla M, Kalra U, Petta A, Sharma S, Shaikh AR, Cavallo L (2025). COCOMAPS 2.0: a web server for identifying, analyzing, and visualizing atomic interactions at the interface of biomolecular complexes. Bioinformatics.

[58] Naveed M, Asim M, Ali A, Amjad S, Majeed MN, Sandrine MNY (2026). Multi-epitope vaccine against nucleoprotein and envelopment polyprotein of Batai orthobunyavirus using molecular docking and molecular dynamics studies. Sci Rep.

[59] Naveed M, Asim M, Aziz T, Majeed MN, Husnain M, Khan AA (2026). Nanoinformatics‐guided inhibition of blaOXA in Staphylococcus nepalensis using cysteine‐conjugated silver nanoparticles. ChemistrySelect.

[60] Laubenbacher R, Adler F, An G, Castiglione F, Eubank S, Fonseca LL (2024). Toward mechanistic medical digital twins: some use cases in immunology. Front Digit Health.

[61] Suleman M, Khan SH, Rashid F, Khan A, Hussain Z, Zaman N (2023). Designing a multi-epitopes subunit vaccine against human herpes virus 6A based on molecular dynamics and immune stimulation. Int J Biol Macromol.

[62] Madeira F, Madhusoodanan N, Lee J, Eusebi A, Niewielska A, Tivey ARN (2024). The EMBL-EBI job dispatcher sequence analysis tools framework in 2024. Nucleic Acids Res.

[63] Naveed M, Asim M, Aziz T, Amjad S, Majeed MN, Jamal SB (2026). Computational design of a multiepitope vaccine targeting VP1 and VP2 capsid proteins of simian virus 40 (SV40) for enhanced immune activation. Open Med.

[64] Bovay A, Fuertes Marraco SA, Speiser DE (2021). Yellow fever virus vaccination: an emblematic model to elucidate robust human immune responses. Hum Vaccines Immunother.

[65] Ponne S, Kumar R, Vanmathi SM, Brilhante RSN, Kumar CR (2024). Reverse engineering protection: a comprehensive survey of reverse vaccinology-based vaccines targeting viral pathogens. Vaccine.

[66] Zhu G, Xu Y, Cen X, Nandakumar KS, Liu S, Cheng K (2018). Targeting pattern-recognition receptors to discover new small molecule immune modulators. Eur J Med Chem.

[67] Brennan K, Bowie AG (2010). Activation of host pattern recognition receptors by viruses. Curr Opin Microbiol.

[68] Hatton AA, Guerra FE (2022). Scratching the surface takes a toll: immune recognition of viral proteins by surface toll-like receptors. Viruses.

[69] Haque MA, Jantan I, Harikrishnan H (2018). Zerumbone suppresses the activation of inflammatory mediators in LPS-stimulated U937 macrophages through MyD88-dependent NF-κB/MAPK/PI3K-Akt signaling pathways. Int Immunopharmacol.

[70] Fadaka AO, Sibuyi NRS, Martin DR, Goboza M, Klein A, Madiehe AM (2021). Immunoinformatics design of a novel epitope-based vaccine candidate against dengue virus. Sci Rep.

[71] Ezzemani W, Windisch MP, Altawalah H, Guessous F, Saile R, Benjelloun S (2023). Design of a multi-epitope Zika virus vaccine candidate–an in-silico study. J Biomol Struct Dyn.

[72] da Silva OLT, da Silva MK, Rodrigues-Neto JF, Santos Lima JPM, Manzoni V, Akash S (2024). Advancing molecular modeling and reverse vaccinology in broad-spectrum yellow fever virus vaccine development. Sci Rep.

[73] Dey J, Mahapatra SR, Raj TK, Kaur T, Jain P, Tiwari A (2022). Designing a novel multi-epitope vaccine to evoke a robust immune response against pathogenic multidrug-resistant Enterococcus faecium bacterium. Gut Pathog.

[74] Mazumder L, Hasan MR, Fatema K, Begum S, Azad AK, Islam MA (2023). Identification of B and T cell epitopes to design an epitope‐based peptide vaccine against the cell surface binding protein of Monkeypox virus: an immunoinformatics study. J Immunol Res.

[75] Sharma AD, Magdaleno JSL, Singh H, Orduz AFC, Cavallo L, Chawla M (2025). Immunoinformatics-driven design of a multi-epitope vaccine targeting neonatal rotavirus with focus on outer capsid proteins VP4 and VP7 and non structural proteins NSP2 and NSP5. Sci Rep.

